# Dynamic mode decomposition of resting-state fMRI revealing abnormal brain region features in schizophrenia

**DOI:** 10.3389/fncom.2025.1742563

**Published:** 2026-01-14

**Authors:** Yaning Wang, Yihong Wang, Xuying Xu, Xiaochuan Pan

**Affiliations:** 1 Institute for Cognitive Neurodynamics, School of Mathematics, East China University of Science and Technology, Shanghai, China; 2 Center for Intelligent Computing, School of Mathematics, East China University of Science and Technology, Shanghai, China

**Keywords:** abnormal brain regions, dynamic mode decomposition (DMD), fMRI, frequency-dependent, schizophrenia

## Abstract

Extracting features from abnormal brain regions in schizophrenia patients’ brain images holds significant importance for aiding diagnosis. However, existing methods remained limited in simultaneously capturing spatiotemporal information. Dynamic mode decomposition (DMD) effectively extracts spatiotemporal features from dynamic systems, making it suitable for time-series signals such as functional magnetic resonance imaging (fMRI) and electrocorticography (ECoG). This study utilized resting-state fMRI data from 68 healthy subjects and 68 schizophrenia patients. The DMD method was employed to extract the mean amplitude of dynamic patterns as features, with feature selection conducted via Least Absolute Shrinkage and Selection Operator (LASSO) regression. A support vector machine (SVM) was further employed to validate the predictive capability of the selected features across subject groups. Based on the LASSO screening, we identified brain regions exhibiting significant inter-group differences in mean amplitude, designated these as abnormal regions, and subsequently analyzed their functional deviations. The DMD method not only provided explicit temporal dynamic representations of brain activity but also supported signal reconstruction and prediction, thereby enhancing feature interpretability. Results demonstrated that DMD effectively extracted mean amplitude features from fMRI data. Combined with LASSO and SVM, it enabled the identification of abnormal brain regions and functional abnormalities in schizophrenia patients. Furthermore, this method captured frequency-dependent signal patterns, with extracted features correlating with both regional activation intensity and functional connectivity. This approach provides novel insights for exploring potential biomarkers of psychiatric disorders.

## 1 Introduction

Schizophrenia is a severe mental disorder associated with abnormalities in brain structure and function (Baker et al., 2014). Functional magnetic resonance imaging (fMRI), with its high spatial resolution, has become a key technique for investigating these neural alterations (Wang et al., 2023; Huda et al., 2024; Wang et al., 2025), as the blood-oxygen-level-dependent (BOLD) signal it provides reflects regional brain energy consumption (Wang et al., 2015; Wang et al., 2021; Peng et al., 2021; Wang et al., 2023).

Currently, the schizophrenia diagnosis relies heavily on clinical interviews and rating scales. This approach faces challenges due to symptom overlap with other disorders (Lu, 2017), ambiguity in diagnostic criteria, and subjectivity in interpretation. There is thus a pressing need for objective, standardized tools to aid diagnosis, and neuroimaging holds significant potential in this regard.

Recently, data-driven algorithms, such as diverse machine learning models (Zhang et al., 2020; Lu and Wang, 2025; Dhongade et al., 2025; Poyraz et al., 2025; Jiang et al., 2025), offer powerful potential for neuroscience by enabling the analysis of complex neural systems and revealing insights that extend beyond traditional methodologies. Among these, dynamic mode decomposition (DMD) is a data-driven method that extracts physically meaningful, low-dimensional spatiotemporal modes from high-dimensional time-series data. Its applicability to nonlinear (Rowley et al., 2009; Tu et al., 2014), high-dimensional (Rich et al., 2016), dynamic and spatiotemporal multiscale (Schmid, 2011; Proctor and Eckhoff, 2015), and sparse systems (Brunton et al., 2015)—properties inherent to neural data—makes it well-suited for neuroscience. Beyond theoretical suitability, DMD has proven effective in extracting coherent modes from diverse datasets, including neurophysiological recordings (Schmid, 2011; Proctor and Eckhoff, 2015; Brunton et al., 2016). When applied to neuroimaging, the spatiotemporal coherent modes extracted by DMD not only complement findings from static analyses (Casorso et al., 2019) and improve neural decoding accuracy (Yoshiyuki et al., 2020), but also predict individual behavioral differences more effectively than temporal and spatial Independent Component Analysis (ICA) (Ikeda et al., 2022). These strengths establish DMD as a promising tool for characterizing the spatiotemporal organization of brain activity.

This study employed DMD on resting-state fMRI data to extract the mean amplitudes of full-band and split-band dynamic modes as brain region features. The Least Absolute Shrinkage and Selection Operator (LASSO) regression was then applied to select key features, which were subsequently validated using a support vector machine (SVM) for their classification efficacy between schizophrenia patients and healthy controls. Results demonstrated that DMD features, obtained through multiple LASSO selections, effectively distinguished between the two groups and accurately localized corresponding brain regions, thereby identifying abnormal brain areas and amplitude deviations in patients. DMD not only revealed the dynamic evolution patterns of brain signals, supporting signal reconstruction and prediction, but also demonstrated correlations between the extracted features and regional brain activation levels. These findings provide an effective tool for exploring abnormal brain function mechanisms in schizophrenia and identifying precise diagnostic and therapeutic targets.

## 2 Materials and methods

### 2.1 Dataset and preprocessing

To investigate the effect of DMD on feature extraction and classification of abnormal brain regions that distinguish schizophrenia patients from healthy subjects, we used the Center of Biomedical Research Excellence (COBRE) dataset. COBRE provided raw anatomical and functional MRI data from 72 patients with schizophrenia and 75 healthy subjects (each group ranged in age from 18 to 65 years). This dataset is publicly available and can be freely downloaded from the public site.^1^ All subjects were screened and excluded if they had a history of neurological disease, intellectual disability, severe head trauma with loss of consciousness for more than 5 min, or substance abuse or dependence within the previous 12 months. Patient diagnostic information was collected using the Structured Clinical Interview for DSM Disorders (SCID).

After removing two dropouts and one subject with missing time points, a subset was selected from the remaining 145 subjects. This selection ensured that the healthy group and the patient group were of equal size and showed no statistically significant differences in age, sex, or dominant hand (*p* > 0.05). Ultimately, data from N=136 subjects were included in the analysis, comprising 68 patients with schizophrenia and 68 healthy controls. Table 1 provides complete demographic information of the dataset used in this study.

**Table 1:** Demographics of subjects in the COBRE dataset.

Features	Health	Schizophrenic	*p*-value
Number	68	68	–
Age(mean ± std)	35.41 ± 11.55	38.54 ± 13.90	0.1584
Sex (male/female)	48/20	54/14	0.2348
Dominant hand (right/non-right)	65/3	59/9	0.0697
Diagnostic Info	/	295.37 ± 0.66	–

The *p*-value for age was obtained using a two-tailed independent samples *t*-test, with the *t*-statistic and degrees of freedom: $t_{\mathrm{age}}(134)=-1.4183$ ; the *p*-values for sex and dominant hand were obtained using a chi-square test, with the corresponding chi-square statistics and degrees of freedom: $\chi_{\mathrm{sex}}(1)=1.4118,\chi_{\text{hand}}(1)=3.2903.$

The resting-state fMRI data were preprocessed using DPABI-DPARSFA (V5.4_230110) (Xia et al., 2013). Preprocessing was performed using a standard procedure that included the following steps: removal of the first 10 time points, temporal layer correction, head motion correction, removal of scalp structures, alignment of structural images to functional images, segmentation, matching images to European templates, denoising, removal of linear drift, adjustment of head motion parameters, filtering, normalization, and smoothing of images, and no global signal regression was performed (Yang et al., 2014). A template of 300 brain regions from ‘Schaefer2018_300Parcels_7Networks_order_FSLMNI152_2mm’ (Schaefer et al., 2018) was used to define the regions of interest, and the BOLD signal time series of 300 regions of interest for each subject were extracted using DPABI-DPARSFA (V5.4_230110), and functional connectivity between brain regions was calculated.

### 2.2 Feature extraction using DMD

#### 2.2.1 Overview of the DMD algorithm

Here, we briefly describe the exact DMD used in this paper, and a more detailed algorithm can be found in previous studies (Tu et al., 2014; Brunton et al., 2016). For a given subject, we have a time series with n nodes: x₁,x₂,…,xn , where the length of each vector is the number of features (such as brain regions) and n is the number of time points sampled, such that $X=[x_{1}\;x_{2}\ldots x_{n-1}],X^{'}=[x_{2}\;x_{3}\ldots x_{n}]$ . Here, X´ denotes the X shifted one step forward in time. And we assume that the transformations of the state of this system are determined by a matrix A, i.e., $X^{'}=\mathrm{AX}$ . The subsequent steps of the algorithm are shown in Table 2 and Brunton et al. (2016).

**Table 2:** Steps of the DMD algorithm.

DMD algorithm
1. Compute the singular value decomposition of X , i.e., $X=\mathrm{US}V^{*}$ , then $X^{'}=\mathrm{AUS}V^{*}$ , where U is the left singular vector matrix, S is the singular value matrix, and $V^{*}$ is the conjugate transpose of the right singular vector matrix.
2. Define $A≜U^{*}\mathrm{AU}=U^{*}X^{'}VS^{-1}$ , $\hat{A}=S^{-1/2}AS^{1/2}$ .
3. Compute the eigenfactorization of $\hat{A}$ : $\hat{A}\hat{W}=\hat{W}\Lambda$ , where $\hat{W}$ is the matrix of eigenvectors and Λ is the diagonal matrix of singular values. The singular values of $\hat{A}$ are the same as the singular values of A , and $W=S^{1/2}\hat{W}$ are the eigenvectors of A used in step 4. These eigenvectors are scaled by $S^{1/2}$ , so they have no unit paradigm.
4. Compute the DMD modes: each column of ϕ is a DMD mode $\varphi_{i}$ , which corresponds to the singular value $\lambda_{i}:\phi ≜X^{'}VS^{-1}W$ .

The core process of the DMD algorithm aims to extract key dynamic patterns from high-dimensional time-series data. First, singular value decomposition (SVD) is applied to the data matrix X to achieve dimensionality reduction and noise removal, thereby identifying the most prominent spatial patterns in brain activity. This establishes an efficient ‘principal coordinate system’ for the complex data. Subsequently, a dynamical operator Ã is defined within this simplified space to capture the core laws governing how the system’s state evolves over time. A characteristic decomposition of Ã then decouples fundamental dynamic components capable of independent, stable oscillation, each characterized by its growth/decay properties and oscillation frequency. Finally, these dynamic components identified in the simplified space are remapped back to the original brain region space, yielding DMD patterns with explicit spatiotemporal structure. Each pattern corresponds to a complete dynamic unit exhibiting coordinated activity across specific brain regions throughout the whole brain, oscillating at a distinct rhythm. This systematic workflow achieves the entire process from data simplification and extraction of dynamic principles to the generation of interpretable neural features.


$$P_{i}={|\varphi_{i}|}_{2}^{2},i=1\ldots M$$ (1)

$$f_{i}=|\text{imag}(\log(\lambda_{i})/\Delta t)/2\pi |,i=1\ldots M$$ (2)

$$x(t)=\phi \Lambda^{t/\mathrm{\Delta t}}\phi^{-1}x(0)$$ (3)

The third step in Table 2 is modified to scale the DMD modes so that the magnitudes of these modes (eigenvectors obtained from the decomposition) can be compared, thus selecting modes with high energy in a manner similar to the power spectrum (Brunton et al., 2016). Equation 1 defines the mode amplitude $P_{i}$ of each mode $\varphi_{i}$ as the square of its vector magnitude; the larger the mode amplitude, the more important the corresponding mode is in the dataset. M represents the number of modes obtained by decomposition. Equation 2 gives the temporal oscillation frequency $f_{i}$ of the mode $\varphi_{i}$ , and Δt is the sampling interval. Equation 3 reconstructs the BOLD signal at time t using the initial state x(0) , the modes ϕ (eigenvectors) and eigenvalues Λ obtained from the decomposition, and it also gives the explicit expression of the resting-state BOLD signal over time.

#### 2.2.2 Data matrix construction and frequency filtering

**Figure 1:**
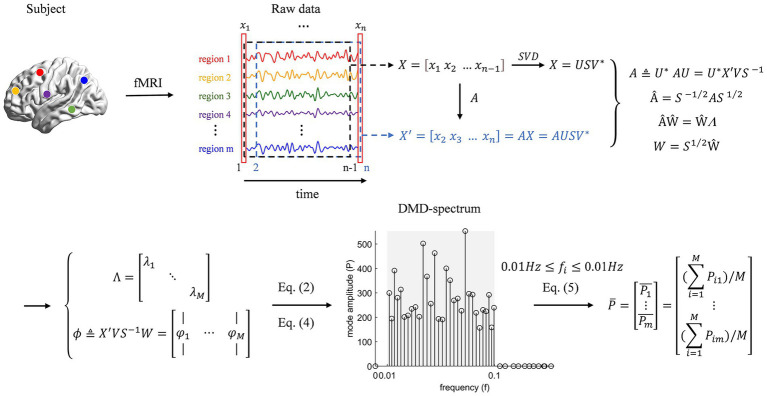
Feature extraction pipeline using dynamic mode decomposition (DMD). The schematic illustrates the process of applying DMD to regional fMRI time series to extract mean amplitude features for each brain region, which are then used for downstream analysis.

The dimension of the time series matrix of regions of interest for each subject obtained after preprocessing is n × m (n = 140, m = 300), where n is the number of time points and m is the number of brain regions. Since the input matrix X of the DMD algorithm should be in the form of features × time, the algorithm is applied to the transpose of the time series matrix for each subject (Figure 1). Each subject was decomposed to obtain 139 modes, and each mode is a complex vector of length m (the number of brain regions).

**Figure 2:**
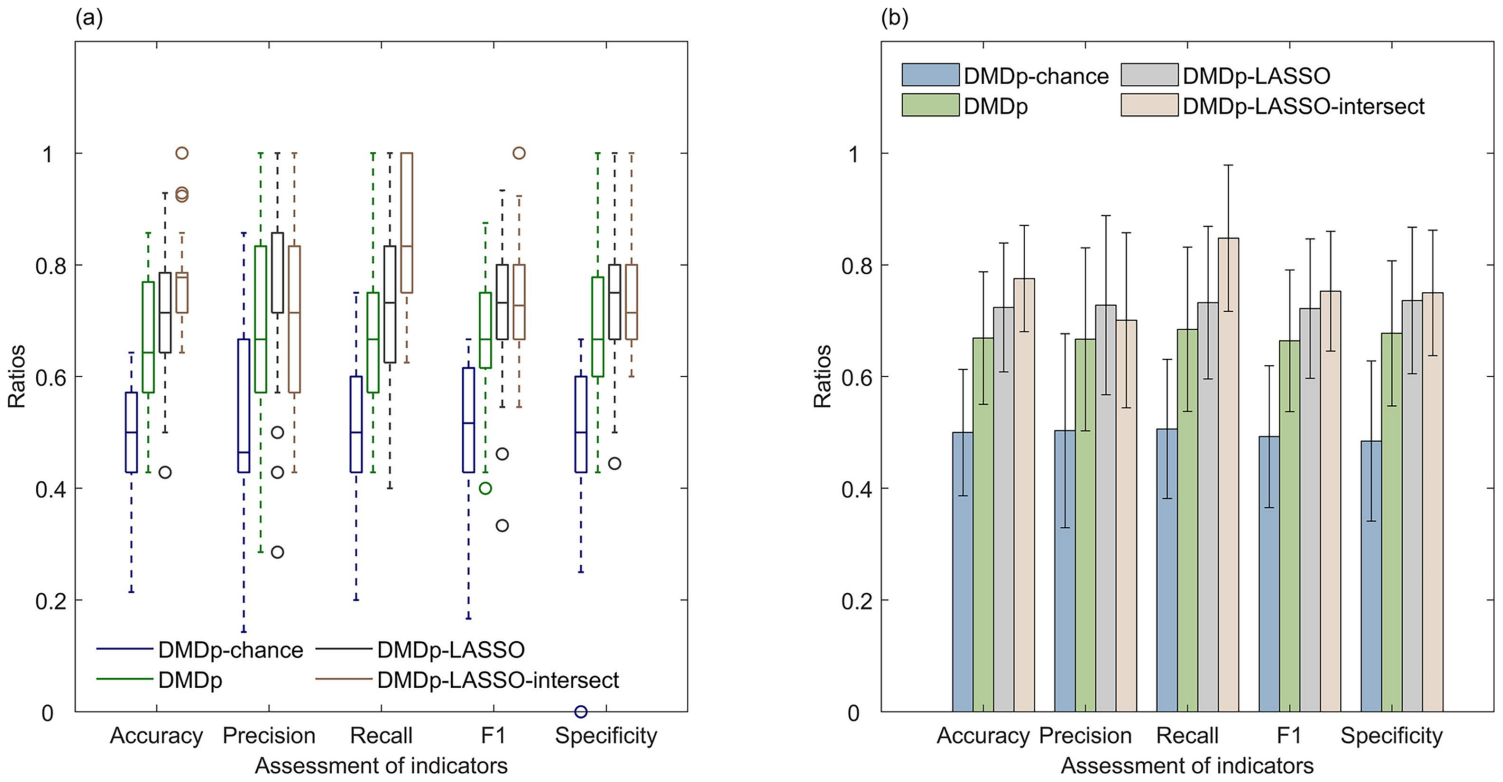
Using DMDp-LASSO-intersect as a classification feature was significantly better than DMDp-chance, DMDp, and DMDp-LASSO. **(a)** Box plots of linear SVM classification indicators for the four cases of DMDp-chance, DMDp, DMDp-LASSO, and DMDp-LASSO-intersect and **(b)** mean bar plots of linear SVM classification indicators.

It was observed in our data that the amplitudes of the DMD modes corresponding to frequencies outside the 0.01–0.1 Hz band were substantially smaller (see Supplementary material in Figures 1, 2). This empirical observation aligns with the established neuroimaging consensus that the blood-oxygen-level-dependent (BOLD) signal fluctuations within the 0.01–0.1 Hz range—termed infra-slow activity—are the most physiologically relevant for resting-state fMRI, as they are robustly linked to spontaneous neural activity and functional network coordination (Fox and Raichle, 2007; Zuo et al., 2010; Sihn and Kim, 2025). Therefore, modes outside this canonical band were considered noise and eliminated. Specifically, frequencies above 0.1 Hz are predominantly contaminated by non-neural physiological noise from cardiac (~1 Hz) and respiratory (~0.2–0.3 Hz) cycles (Bancelin et al., 2023), while very-low-frequency fluctuations below 0.01 Hz may be confounded by scanner drift or ultra-slow physiological processes (Smith et al., 1999). Applying this standard frequency filter enhances the signal-to-noise ratio for interpreting intrinsic brain dynamics.

There were M=62or64 modes per subject in the 0.01–0.1 Hz band, with complex modes appearing in conjugate pairs, and each mode having a corresponding frequency [defined in Equation 2].


$$P_{\mathrm{ij}}=|\varphi_{\mathrm{ij}}|2,i=1\ldots M;j=1\ldots m$$ (4)

$${\text{feature}}_{j}=\sum_{i=1}^{M}|\varphi_{\mathrm{ij}}|2/M,j=1\ldots m$$ (5)

#### 2.2.3 Feature extraction and computation

$P_{i}$ characterizes the strength of the spatial pattern of activity corresponding to that mode $\varphi_{i}$ in the overall signal (Brunton et al., 2016), and then $P_{\mathrm{ij}}$ [defined in Equation 4] reflects the signal strength of the corresponding pattern $\varphi_{i}$ in the respective brain region numbered j. Since the modes are complex vectors, using the amplitudes of the modes is a good way to avoid dealing with the real and imaginary parts of the complex numbers. $P_{\mathrm{ij}}\;$ were calculated for every mode i and every brain region j, and then the $P_{\mathrm{ij}}$ of all modes of each brain region were averaged according to the number of modes M to obtain the corresponding average signal intensity ${\text{feature}}_{j}$ [defined in Equation 5] of each brain region numbered j, which we used as the mean amplitude characteristics of the brain regions in the whole frequency band extracted by DMD for each subject.

In addition, to explore and take advantage of the fact that the DMD algorithm can extract both temporal and spatial information, we also extracted the features of each subject’s sub-frequency bands. That is, 0.01–0.1 Hz is divided into a number of frequency bands, then $P_{\mathrm{ij}}$ of all mode components in each frequency band were averaged by the number of modes in that band, and the mean values of all bands were spliced together to obtain the average signal intensity of each brain region in different frequency bands, which is used as the sub-frequency band features extracted by DMD in each subject to explore the relationship between abnormal signals and frequencies in brain regions of schizophrenic patients.

### 2.3 LASSO-based feature selection combined with SVM-based classification

To evaluate the classification capability of extracted features for subjects (patient/healthy), we employed SVM for classification modelling. By comparing classification performance across different feature sets, we validated the effectiveness of the features. Before inputting the features into the SVM, the mean amplitude features of the brain regions (full-band and sub-band) obtained from each subject were subjected to LASSO regression to exclude the unimportant feature variables. The definition of LASSO regression is given in Equation 6, where y is the target variable, an N-dimensional vector; X is the feature matrix of size N × p, where N is the number of samples and p is the number of features; *β* is the vector of regression coefficients of size p-dimensional; and γ is the regularization parameter, which controls the strength of the L1 regularization. LASSO regression was fitted using 10-fold cross-validation with the initialization parameter γ=0.2 . For each fold, feature selection via LASSO was performed solely on the training set. The selected features were subsequently tested on the corresponding test set. The effectiveness of the classifier was estimated by calculating five indicators: accuracy, precision, recall, F1 score, and specificity, which are commonly used to evaluate the performance of classification models. During classification, we subjected the dataset to three rounds of ten-fold cross-validation, yielding 30 sets of performance indicators. We then ran 1,000 permutation tests of the classification indicators for different features to see if they were statistically significantly different.

To validate the efficacy of features selected via LASSO in distinguishing between patient and healthy groups, we compared the SVM classification performance of three sets of brain region mean amplitude features: those obtained with randomly perturbed training labels, those without LASSO selection, and those after a single round of LASSO selection. Furthermore, as a single run of LASSO regression carries inherent randomness, to achieve stable feature selection outcomes, we also compared the SVM classification performance of intersecting brain region features selected through 1,000 LASSO regressions performed during three independent runs of ten-fold cross-validation (i.e., 30 iterations of LASSO regression). The four feature sets underwent three-fold cross-validation under identical training/test set divisions. By comparing their classification performance, we comprehensively evaluated the robustness and discriminative power of the DMD-extracted mean amplitude features and the feature selection methods.

Given that a single run of LASSO regression may yield unstable results due to the inherent randomness of the algorithm—theoretical studies indicate risks of prematurely selecting spurious variables (Su, 2018) and an inclination toward over-selecting features (Liu et al., 2023)—to obtain an absolutely robust feature subset, we performed 1,000 independent LASSO regressions. We strictly restricted the final feature set to include only those consistently selected across all 1,000 runs. This screening criterion overcomes the instability of variable selection in high-dimensional data, thereby ensuring the final feature set used for classification exhibits high reproducibility and statistical reliability.


$$\hat{\beta }=\arg\min_{\beta }(({\left\|y-\mathrm{X\beta}\right\|}_{2}^{2}+\gamma {\left\|\beta \right\|}_{1})/2N)$$ (6)

### 2.4 Analysis of abnormal brain regions in patients with schizophrenia

The mean amplitude of both full-band and sub-band brain region features was mapped to corresponding brain regions, as each feature constituted a 300-dimensional vector corresponding to 300 distinct brain regions. To fully utilize the available data in exploring abnormal brain regions in schizophrenia patients, we applied LASSO regression to the mean amplitude features of all subjects’ brain regions, identifying key features critical for distinguishing patients from healthy controls. However, considering the randomness in variable retention during each LASSO regression, we performed 1,000 independent LASSO regression analyses on the full-band and sub-band mean amplitude features to identify consistently selected brain region features—that is, features with robust dominant roles in classification regardless of the number of regressions performed. We then identified the consistently retained brain regions by evaluating their intersections across these analyses. Subsequently, we conducted 1,000 permutation tests on the intersecting brain region features between the healthy and patient groups. Brain regions showing significant differences at a significance level of *α* = 0.05 were defined as abnormal brain region features in schizophrenia patients.

We also calculated the average regression coefficients $\bar{\beta }$ of these abnormal brain region features across 1,000 LASSO regressions. The LASSO regression coefficients can reflect the contribution and influence of each feature on the prediction results. Specifically, positive values ( $\bar{\beta }i>0$ ) indicate that the feature is positively correlated with the target variable, negative values ( $\bar{\beta }i<0$ ) indicate a negative correlation, and the magnitude of the absolute value $\bar{\beta }i$ indicates the degree of influence. By analyzing the average regression coefficients for each abnormal brain region, we can understand the direction of deviations of abnormality and its severity in the abnormal brain regions of schizophrenic patients.

## 3 Experimental results

### 3.1 Effectiveness of using full-band brain region mean mode amplitudes as features and performing LASSO regression

We compared the SVM classification performance of four distinct sets of brain region mean amplitude features: features derived using randomly perturbed training labels (DMDp-chance), features without LASSO selection (DMDp), features selected through a single round of LASSO regression (DMDp-LASSO), and intersecting brain region features (DMDp-LASSO-intersect) identified by LASSO regressions performed during three independent runs of ten-fold cross-validation.

The results showed (Figure 2; Table 3) that at the significance level of α = 0.05, the classification effects of DMDp, DMDp-LASSO and DMDp-LASSO-intersect were significantly better than that of DMDp-chance for all indicators, and the classification effect of DMDp-LASSO-intersect was significantly better than that of DMDp for accuracy, recall, F1 score, and specificity. The classification effect of DMDp-LASSO-intersect was significantly better than that of DMDp-LASSO for accuracy and recall. Therefore, the use of mean brain region amplitudes as classification features, LASSO for feature selection, and the intersection from multiple LASSO regressions as a key discriminant proves to be an effective strategy for distinguishing the two groups.

**Table 3:** Permutation test *p*-values for the linear SVM classification indicators for DMDp-chance, DMDp, DMDp-LASSO and DMDp-LASSO-intersect.

Objects of the permutation test (*p*_value)	Accuracy	Precision	Recall	F1	Specificity
DMDp vs. DMDp-chance	9.90E-03**	9.90E-03**	9.90E-03**	9.90E-03**	9.90E-03**
DMDp vs. DMDp-LASSO	7.92E-02	1.58E-01	2.57E-01	3.96E-02*	9.90E-02
DMDp vs. DMDp-LASSO-intersect	9.90E-03**	4.46E-01	9.90E-03**	9.90E-03**	2.97E-02*
DMDp-LASSO vs. DMDp-chance	9.90E-03**	9.90E-03**	9.90E-03**	9.90E-03**	9.90E-03**
DMDp-LASSO vs. DMDp-LASSO-intersect	3.96E-02*	6.14E-01	1.98E-02*	3.27E-01	6.34E-01
DMDp-LASSO-intersect vs. DMDp-chance	9.90E-03**	9.90E-03**	9.90E-03**	9.90E-03**	9.90E-03**

*Indicates *p*-value < 0.05; **indicates *p*-value < 0.01; ***indicates *p*-value < 0.001.

### 3.2 Abnormal brain region activity in schizophrenic patients

A total of 28 brain regions were retained in each of the 1,000 LASSO regression analyses of the mean amplitude characteristics of brain regions across the frequency band. At the α=0.05 significance level, 22 of the 28 brain region features were significantly different between health and patient groups. We considered the 22 brain region features with significant differences to be the abnormal brain region features that play an important role in the categorization and discriminate schizophrenics from the healthy population.

**Figure 3:**
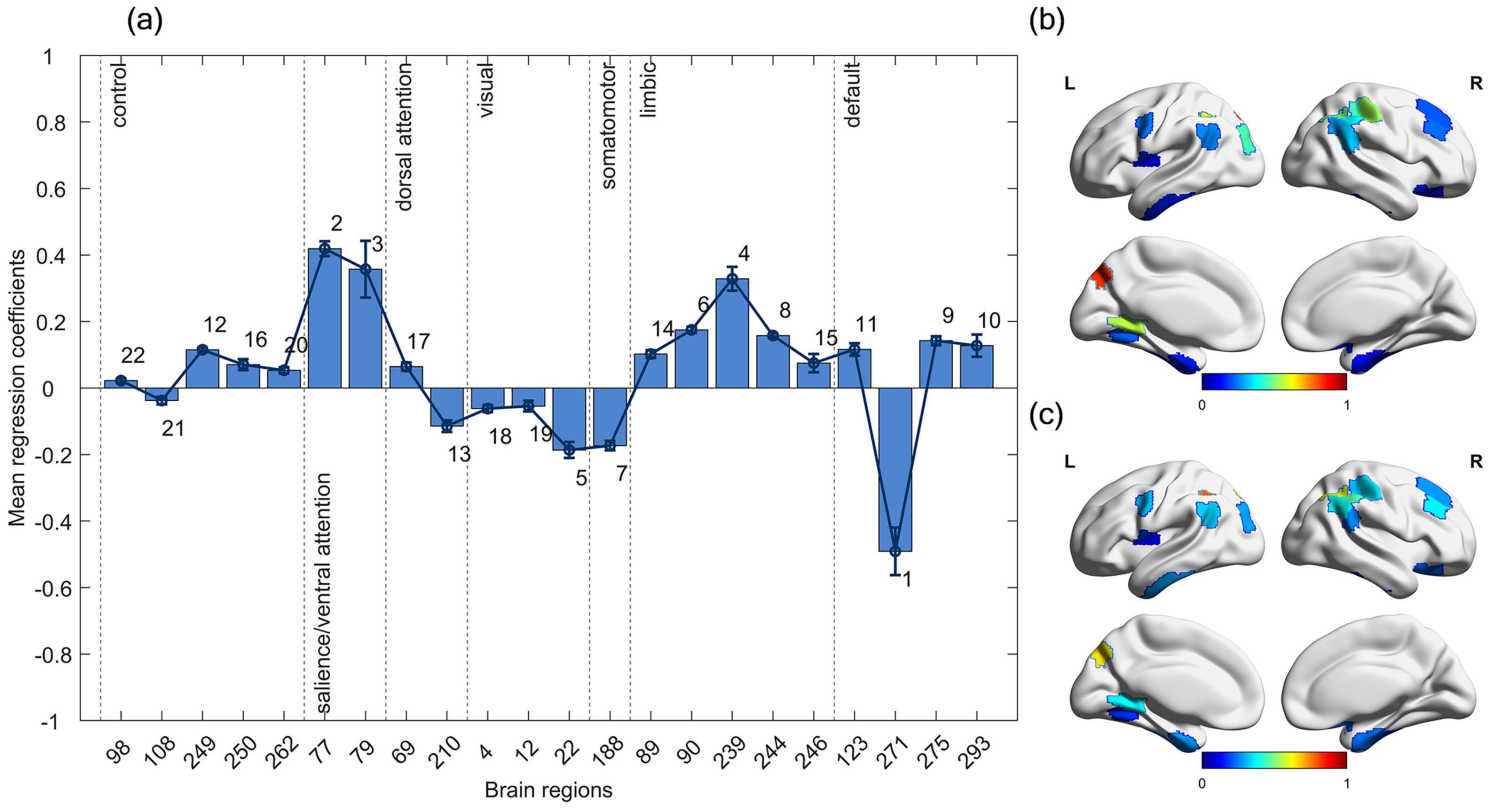
Brain regions in several functional networks had the same direction of deviations of abnormality. **(a)** Mean regression coefficients for abnormal brain region features in the full frequency band. The associated number ranks each feature’s importance for classification (1 = most influential), based on $|\overset{¯|}{\beta_{i}}$ (mean absolute regression coefficient). **(b,c)** Visualization of the mean amplitudes for the 22 abnormal brain regions in **(b)** the healthy group and **(c)** schizophrenia patients.

In Figure 3a, we plotted bar graphs of the mean regression coefficients $\bar{\beta }$ and line graphs with error bars for these 22 brain regions (The brain regions involved and their numbers are as follows: 98-'parietal’, 108-'precuneus’, 249-'parietal’, 250-'parietal’, 262-'lateral prefrontal cortex’, 77-'frontal operculum insula’, 79-'frontal operculum insula’, 69-'precentral ventral’, 210-'posterior’, 4-'visual’, 12-'visual’, 22-'visual’, 188-'somatomotor’, 89-'temporal pole’, 90-'temporal pole’, 239-'orbital frontal cortex’, 244-'temporal pole’, 246-'temporal pole’, 123-'parietal’, 271-'parietal’, 275-'parietal’, 293-'dorsal medial prefrontal cortex’) using the functional brain network as a partition. In the SVM classification, we labeled patients as 1 and healthy subjects as 0. Therefore, the positive coefficients of the LASSO regression indicate that the mean amplitudes of the patient group in that brain region are higher than that of the healthy group, and the negative coefficients indicate that the mean amplitudes of the patient group in that brain region are lower than that of the healthy group, and the absolute magnitude indicates how abnormal the brain region is relative to the healthy group. Figures 3b,c visualizes the mean amplitude magnitudes of the 22 abnormal brain regions for the healthy group and the patient group.

The mean amplitudes of brain regions associated with the parietal, lateral prefrontal cortex, frontal operculum insula, precentral ventral, temporal pole, orbital frontal cortex, and dorsal medial prefrontal cortex were higher in the patient group than in the healthy group; and the mean amplitudes of brain regions associated with the precuneus, posterior, visual, somatomotor, and parietal were lower in the patient group than in the healthy group. Abnormal brain regions belonging to the salience/ventral attention network and limbic network had higher mean amplitudes in the patient group than in the healthy group; abnormal brain regions belonging to the visual network, and somatomotor network had lower mean amplitudes in the patient group than in the healthy group. According to the ranking of the degree of abnormality, the top ten abnormal brain regions were distributed in the salient/ventral attention network, the visual network, the somatomotor network, the limbic network, and the default mode network.

### 3.3 Classification effects and abnormal brain regions using sub-band mean amplitude features

**Figure 4:**
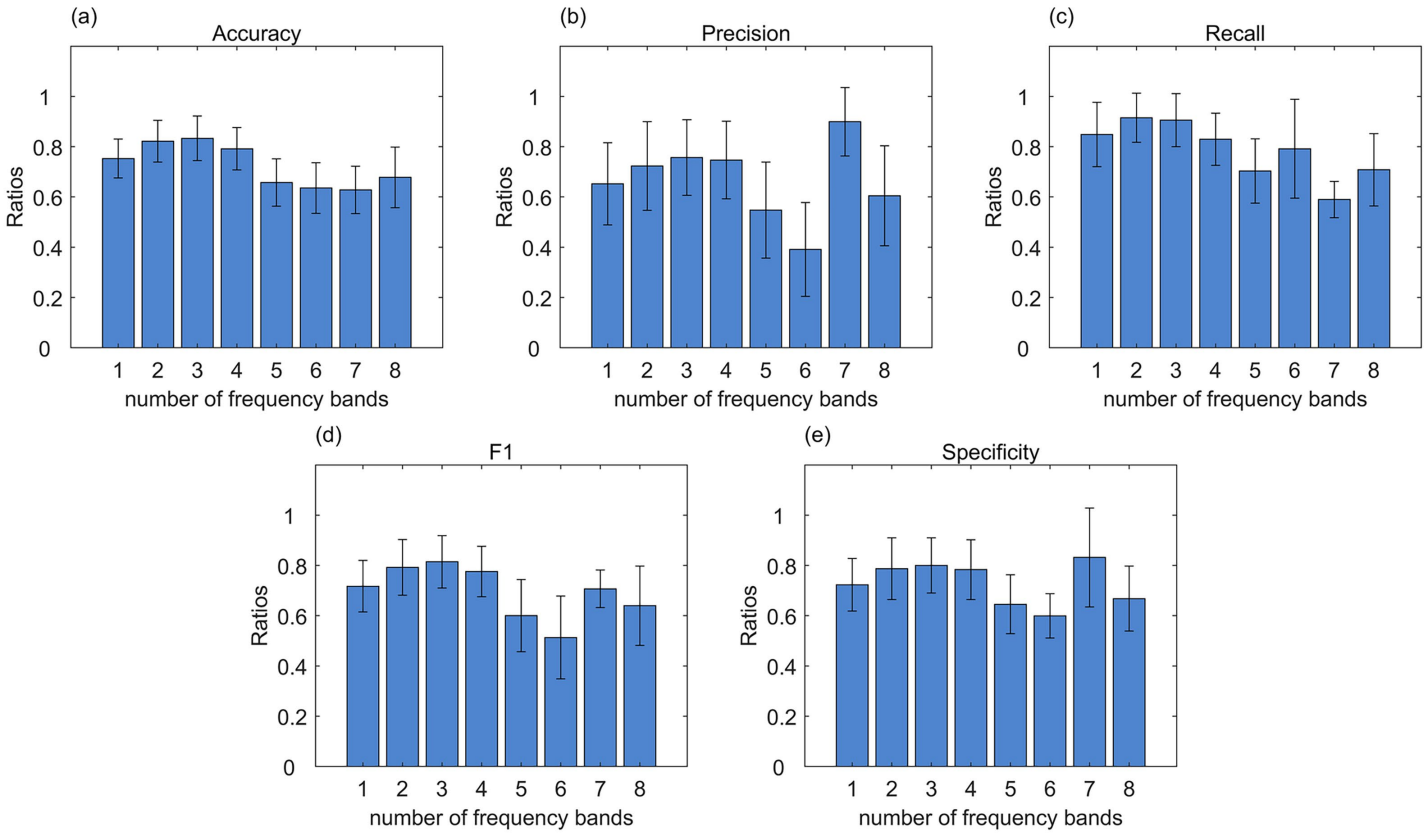
Classification effects can be significantly improved by using mean amplitude features of brain region modes averaged over three frequency bands. **(a–e)** Shows the bar graphs of frequency band comparison for the five indicators. The horizontal coordinates indicate the number of frequency bands; in particular, 1 indicates that there is only one band, i.e., no sub-banding.

We averaged the 0.01–0.1 Hz bands into *k* bands (*k*∈*[2,8]*), and spliced the mean amplitude features of the different bands together horizontally, so that m∗k features were available for each subject. At a significance level of α=0.05 , classification results obtained by dividing features into either two or three bands were significantly superior to those without segmentation. Furthermore, the mean classification indicators achieved when dividing into three bands demonstrated better performance across most indicators. As shown in Figure 4, the improvement in the mean value of the classification indicators reached saturation when divided into three frequency bands. In this paper, we chose to divide it into three frequency bands ([0.01, 0.04] Hz, [0.04, 0.07] Hz, [0.07, 0.1] Hz) to explore the relationship between signal abnormalities and signal frequency in brain regions of schizophrenic patients. Each frequency band corresponded to 300 brain region features, and after splicing the features of the three frequency bands, each subject had a total of 900 brain region features. Similarly, the LASSO regression step was repeated 1,000 times, and a total of 14 brain region features were retained in each regression: 10 in the [0.01, 0.04] Hz frequency band, 2 in the [0.04, 0.07] Hz frequency band, and 2 in the [0.07, 0.1] Hz frequency band. All the 14 brain region features differed significantly between the health and patient groups.

11 of the 14 brain regions (The brain regions involved and their numbers are as follows: lowf-262-'lateral prefrontal cortex’, lowf-69-'precentral ventral’, lowf-22-'visual’, lowf-188-'somatomotor’, lowf-89-'temporal pole’, lowf-246-'temporal pole’, midf-239-'orbital frontal cortex’, higf-90-'temporal pole’, lowf-123-'parietal’, lowf-271-'parietal’, lowf-275-'parietal’) appeared in the full frequency screening of abnormal brain regions, and the features of the remaining 3 brain regions (The brain regions involved and their numbers are as follows: midf-97-'parietal’, lowf-65-'posterior’, higf-92-'temporal pole’) that did not appear differed significantly between health and patient groups.

**Figure 5:**
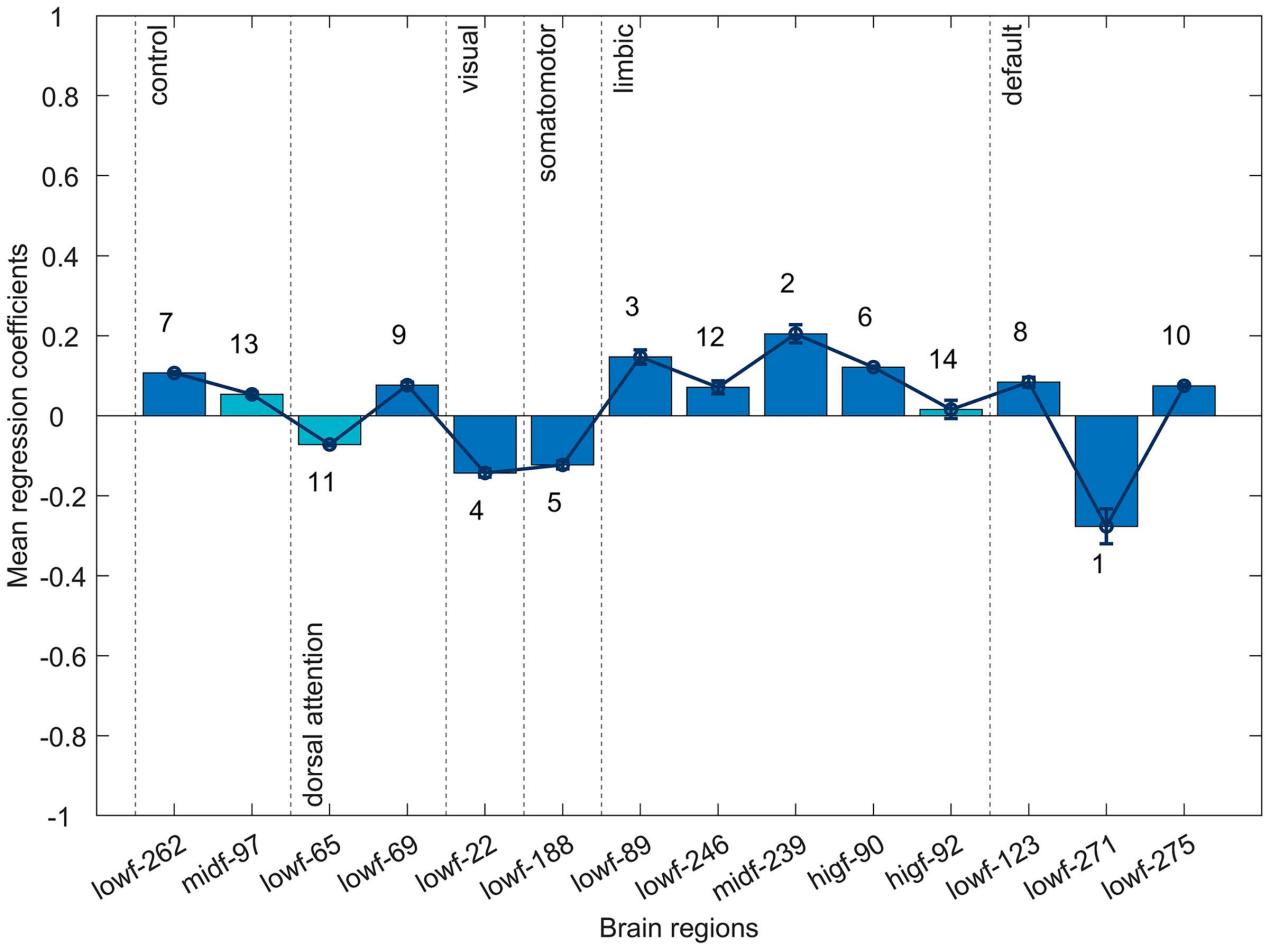
Newly identified abnormal brain region features in sub-frequency band analysis. Bar graph of the mean regression coefficients for features identified within three sub-bands: low- (lowf, [0.01, 0.04] Hz), mid- (midf, [0.04, 0.07] Hz), and high-frequency (higf, [0.07, 0.1] Hz). Features highlighted in cyan were newly detected and not found in the full-band analysis.

97-parietal in the control network, 65-posterior in the dorsal attention network, and 92-temporal pole in the limbic network, the mean amplitudes of the full frequency bands of these brain regions did not differ significantly between health and patient groups, but after band interval slicing these brain region features in the corresponding sliced frequency bands were significantly different between the two groups. That is, schizophrenic patients have abnormal mean signal intensities in brain regions 65 in the [0.01, 0.04] Hz frequency band, 97 in the [0.04, 0.07] Hz frequency band, and 92 in the [0.07, 0.1] Hz frequency band. In addition, to explore the degree of influence of abnormal brain region signals in different frequency bands, we also plotted the bar graph of the average regression coefficients of the 14 brain region features and the line graph with error bars with the network as a subdivision in Figure 5. Abnormal brain regions belonging to the control network and limbic network had higher mean amplitudes in the patient group than in the healthy group; abnormal brain regions belonging to the visual network, and somatomotor network had lower mean amplitudes in the patient group than in the healthy group.

### 3.4 Effectiveness of using modes obtained by DMD for reconstruction and prediction the BOLD signals

**Figure 6:**
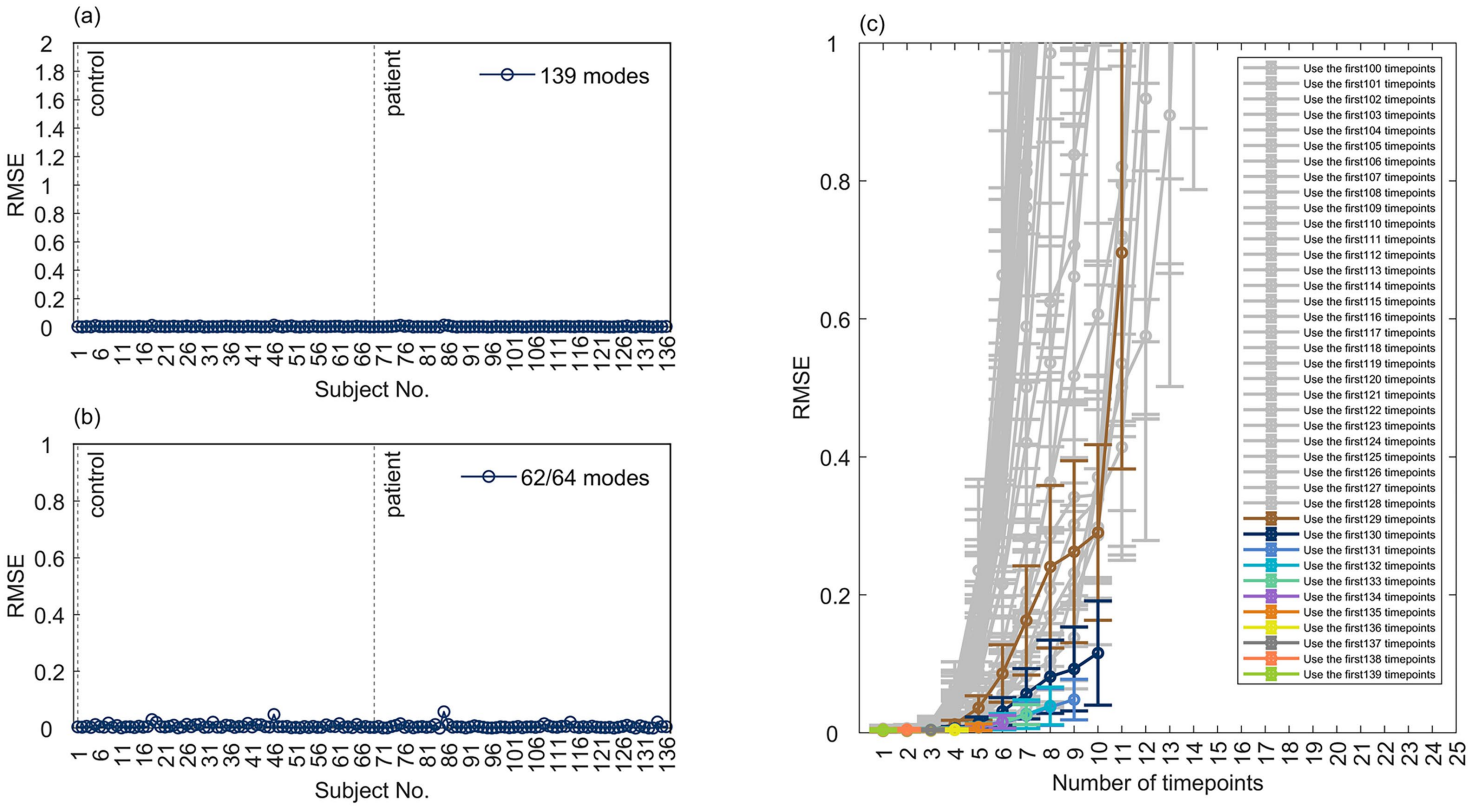
DMD enables effective BOLD signal reconstruction and prediction. **(a,b)** Reconstruction performance using **(a)** all decomposed modes versus **(b)** noise-filtered modes per subject. **(c)** Mean prediction error (RMSE) versus the number of future time points forecasted, based on decompositions starting from varying initial segments of the time series.

The values of the BOLD signals used to perform the DMD ranged in the [−50, 50] interval but were concentrated in the [−10, 10] interval. As shown in Figures 6a,b, the overall mean RMSE of the reconstruction using all 139 modes obtained by decomposing the BOLD signals for each subject averaged 0.0044, and the overall mean RMSE of the reconstruction using the 62/64 modes with frequencies in the interval [0.01, 0.1] Hz for each subject averaged 0.0068. The good reconstruction results indicate that within the time window of the decomposition, Equation 3 can explicitly express the change in activity of the brain BOLD signal for each time point.

The prediction mean RMSE series for the 136 subjects were visualized with different colored lines with error bars after removing outliers, as shown in Figure 6c. The vertical coordinates of the error plots were restricted to the interval [0,1], and we considered an RMSE greater than 1 to indicate poor prediction and focused only on when it was less than 1. When the time series of the first 130–139 time points were used for prediction, the prediction mean RMSE for each remaining time point was kept below 0.2; when the time series of the first 131–139 time points were used for prediction, the prediction mean RMSE for each remaining time point was kept below 0.1. The general effect of predicting the remaining time points using the 100–139 time points is that the predictions of the first 5 remaining time points are very effective, with mean RMSEs below 0.3, and from the 6th time point onward, the prediction effect gets worse and worse, and the mean RMSE shows an exponential growth. The original features extracted using the DMD can stably predict the changes in the BOLD signal activity for the following 5 unknown time points (10 s), and the more time points used in the decomposition, the better the overall prediction effect.

## 4 Discussion

### 4.1 Reproducibility of the results and correlation with existing results

To test the reproducibility of the results, we also used a template of 100 brain regions from ‘Schaefer2018_100Parcels_7Networks_order_FSLMNI152_2mm’ (Schaefer et al., 2018) to define the regions of interest and obtained consistent results (see Supplementary material).

Schizophrenia is characterized by cognitive, affective, and behavioral dysregulation, often accompanied by cognitive and social dysfunction. Functional studies have found abnormalities of activation within brain regions and abnormalities of connectivity between brain regions in patients, but these may vary depending on whether the patient exhibits negative or positive symptoms (Dabiri et al., 2022). The brain regions identified as abnormal in this study may represent the functional abnormalities described above at the level of brain networks. Further comparison with existing research indicates that regions exhibiting higher average amplitude than the healthy control group may correspond to enhanced functional connectivity within the patient’s networks or increased activation levels in the respective brain areas. This provides a novel perspective for elucidating the neural mechanisms underlying brain functional alterations in schizophrenia.

In this study, abnormal brain regions in the full frequency band had the same direction of deviations of abnormality in the salience/ventral attention network, limbic network, visual network, and somatomotor network; and abnormal brain regions in three frequency bands had the same direction of deviations of abnormality in the control network, the limbic network, visual network, and somatomotor network. These spatially consistent patterns of abnormality provide crucial clues for further understanding the brain dysfunction associated with schizophrenia.

Building upon these spatially consistent deviations, we can discern a more fundamental, system-level regularity in the brain activity of schizophrenia patients: patients with schizophrenia consistently demonstrate heightened activity within networks involved in internal motivation, emotion, and self-referential processing (such as the salience/ventral attention network, limbic network, and control network). Conversely, networks responsible for perceiving and acting upon the external world (such as the visual network and somatomotor network) show reduced activity. This functional disconnection pattern of ‘internal hyperactivity and external hypoactivity’ provides crucial clues for understanding the core pathophysiological mechanisms of the disorder. This is not a random connectivity aberration but likely reflects a fundamental cooperative dysfunction in the brain’s integration of internal states and external reality. This finding resonates with prevailing theories in the field: firstly, the hyperactivity of the salience network—a central hub for information filtering—supports the ‘salience network dysfunction’ hypothesis (Mantonakis et al., 2024; Bolton et al., 2020), potentially representing the initial link driving individuals to misinterpret internal thoughts as external stimuli, thereby triggering positive symptoms. Secondly, this systemic imbalance between internally directed and externally directed network clusters provides macro-level corroboration for schizophrenia’s ‘brain network disconnection’ theory (Friston et al., 2016; Harikumar et al., 2023). This posits that disrupted mechanisms of functional integration and disconnection between large-scale brain networks underlie the broad spectrum of clinical symptoms ranging from thought disorder to psychomotor disturbances. Consequently, the characteristic patterns identified in this study reveal a potential cross-network pattern of systemic functional disengagement in schizophrenia, offering a novel neurobiological perspective for a unified explanation of its complex symptom spectrum.

Moreover, the abnormal signals in the patient’s brain regions are frequency dependent, and the averaging of the full frequency bands will lose this part of the information. The appropriate inclusion of frequency information can significantly improve the classification effect, and can also find the patients’ characteristic brain regions with abnormal signals hidden among different frequency bands.

The core idea of our sub-band analysis—extracting finer-grained information obscured by broad-band averaging through multi-scale decomposition—aligns with the well-established methodological framework of multi-resolution analysis prevalent in signal processing and pattern recognition. This framework typically involves decomposing data into components at different scales or resolutions, performing feature extraction and analysis on each sub-band, and synthesizing these multi-level insights for a more robust and discriminative model, as demonstrated in domains such as medical image analysis (Moorthy et al., 2024). Applying this effective paradigm to resting-state fMRI, our study aims to isolate and characterize frequency-specific neural oscillatory patterns that may carry distinct pathophysiological significance but are smoothed over in full-band analysis. This approach provides a novel analytical perspective for investigating potential frequency-selective alterations in large-scale network dynamics in schizophrenia.

### 4.2 Comparison of DMD with other feature extraction methods

In biomedical signal analysis, adaptive decomposition techniques, including wavelet-based methods (e.g., the Empirical Wavelet Transform, EWT) (Khan and Pachori, 2025; Khan and Pachori, 2024; Khan and Pachori, 2022) and Empirical Mode Decomposition (EMD) (Agrawal et al., 2024), have proven effective for extracting discriminative, time-frequency-based features from diverse signals for automated diagnosis. Extending this paradigm of data-driven feature extraction to functional neuroimaging, our study employs DMD, which offers a distinct advantage for analyzing brain activity: it is fundamentally designed to characterize spatiotemporal dynamics. Unlike the purely time-frequency representations generated by the aforementioned techniques, DMD extracts coherent spatiotemporal modes that directly capture the evolving patterns of large-scale neural coordination, making it particularly suited for investigating the altered brain dynamics in disorders such as schizophrenia.

In the context of fMRI data, methods such as PCA and ICA have been used to identify brain networks that form functional connections (Viviani et al., 2005; Ulfarsson and Solo, 2007; Smith et al., 2012; Calhoun et al., 2009; Eavani et al., 2015; Hirayama et al., 2016). However, these methods are static because they treat consecutive time points of a multivariate time series as independent observations. The DMD algorithm, on the other hand, is dynamic and combines the well-characterized advantages of two of the most powerful data analysis tools in use today: power spectral analysis in time and principal component analysis in space, which allows for a much clearer separation of modes that are mixed in time and space (Brunton et al., 2016; Casorso et al., 2019), leading to better extraction of features of dynamic data. Some of the functional connectivity changes found by dynamic analysis are not found in static connectivity analysis (Damaraju et al., 2014). Furthermore, both PCA and ICA treat signals as linear combinations of feature time series, which does not match the inherent nonlinear neural dynamics of the brain. Although the process of identifying DMD modes and eigenvalues is also purely linear, the system itself can be nonlinear, and the link between the Koopman operator and DMD can demonstrate that nonlinear systems can be described by a set of modes and eigenvalues (Rowley et al., 2009; Tu et al., 2014). Thus, DMD provides a new perspective that allows us to understand and analyze the complex dynamics of brain activity in greater depth, which is important for revealing the deeper mechanisms of brain function and improving the diagnosis and treatment of psychiatric disorders.

To validate the effectiveness of mean amplitudes extracted from brain regions using DMD as features, we employed several feature extraction methods for comparative analysis. These additional features included the Gram matrix of DMD-based features (DMD-Gram) (Ikeda et al., 2022), the Gram matrix of PCA-based features (PCA-Gram), the Gram matrix of ICA-based features (ICA-Gram), and spatial node DMD features (snDM) (Fukuma et al., 2024). It should be noted that the Gram matrix cannot be used with LASSO regression due to the equal status of each component. Under the same training/test set split, we performed classification using a linear SVM and carried out three rounds of ten-fold cross-validation. Finally, we conducted 1,000 permutation tests to evaluate the significance of the classification performance. The results showed that the classification performance of DMD-LASSO did not differ significantly from that of DMD-Gram, PCA-Gram, or snDM-LASSO, whereas all four methods (DMD-LASSO, DMD-Gram, PCA-Gram, and snDM-LASSO) significantly outperformed ICA-Gram (see Supplementary material in Figure 3; Supplementary material in Table 1).

### 4.3 Data reconstruction and prediction

This paper investigated the better properties of the DMD algorithm for reconstructing the decomposed signal and predicting the unknown signal. The DMD algorithm decomposes the signal and gives the modes and eigenvalues as well as the explicit expressions of the signal at each point of time. Inputting a value at a different point in time will give the value of the BOLD signal at that point in time, which will allow the reconstruction of the decomposed signal and the prediction of the unknown signal. The original BOLD signal data contains some noise, and reconstructing the data using modes in a certain frequency band may also be an effective method for denoising the BOLD signal. Using the filtered modes can predict the unknown signal for the next 5 time points more stably, and may be able to provide more accurate interpolation for fMRI datasets with missing time-point data. This further illustrates the complexity and nonlinearity of the brain, i.e., the dynamic model constructed by DMD only captures brain activity within a few time steps outside the decomposition window.

### 4.4 The sequence correlation matrix of the DMD modes is highly similar to the functional connectivity (FC) matrix

In the present study, we further found that the sequence correlation matrix (phiC) between modes obtained from the decomposition of each subject was approximately equal to the functional connectivity (FC) matrix of its original signal. Specifically, the dimension of the matrix formed by all the modes of each subject was 300 × 62/64, and by calculating the Pearson correlation coefficients of the mode sequences in each brain region, we obtained a 300 × 300 mode sequence correlation matrix; whereas the correlation matrix of the original BOLD signal time series formed the functional connectivity matrix. We calculated the Pearson correlation coefficients and RMSEs between phiC and FC using 139 and 62/64 modes, respectively. The mean correlation coefficients for phiC and FC were 0.9860 (using 62/64 modes) and 0.9893 (using 139 modes), respectively, and the mean RMSEs were 0.0464 (using 62/64 modes) and 0.0401 (using 139 modes) (see Supplementary material in Figure 4).

Upon probing, the same property is found on other fMRI datasets and synthetic datasets consisting of periodic sine waves without noise. FC, which usually measures the Pearson correlation of the time course between different brain regions, can represent the temporal synchronization between different brain regions. And when reconstructed according to the DMD modes, the signal value at each time point consists of a time-dependent linear combination of all the modes, and phiC actually represents the synchronization of activity between brain regions as well. FC and phiC characterize the same thing, but the characterization of phiC is a bit freer. By choosing specific modes to build phiC, we can explore the connectivity of these modes in brain regions.

### 4.5 Limitations and future directions

First, the modes obtained by DMD have significant individual variability. Using the DMD features of one subject to reconstruct its own time series works well, but when used for another subject, the RMSE increases to the order of 10^3, which is not effective. Therefore, individual characterization is not representative of the whole group, and the use of group-shared characteristics can reflect the group characteristics to some extent.

Second, the COBRE dataset used in this study lacks information on the stage of onset of schizophrenia in patients with schizophrenia. The varying degrees of brain lesions in patients with schizophrenia at different stages of onset may affect the effectiveness of classifiers and the exploration of abnormal brain regions (Shen et al., 2023; Gong et al., 2016). Beyond the absence of illness stage information, a further data-driven limitation pertains to the quantification of clinical symptoms. As the COBRE dataset employed did not include standardized quantitative scores for clinical symptom severity (such as the PANSS scale), we were unable to establish associations between the extracted DMD features and specific clinical symptom dimensions within this analysis. This limitation at the data level implies that the present study primarily validates the sensitivity of the DMD method in characterizing disease-related brain dynamics, without yet revealing connections between these features and individual clinical phenotypes. Future research must systematically examine the correlations between these dynamic features and symptom severity, cognitive function, or treatment response within prospective datasets incorporating multidimensional clinical assessment data (Métivier and Dollfus, 2025). This constitutes a critical step in evaluating their potential as clinical biomarkers (Schmitt et al., 2017; Wang et al., 2023).

Finally, due to the nonlinearity and variability of brain activity, the DMD model is not good at predicting future time series for BOLD signal, and in this study was only able to remain stable predictions over 5 timesteps. This limits the temporal scalability of the features obtained by the algorithm. However, the features can still characterize the main resting-state activities of the brain better, provided that enough time-point information is decomposed.

Moreover, it must be explicitly stated that as a cross-sectional observational study, the findings reveal stable associations between schizophrenia and specific brain dynamics patterns. However, these are inherently correlational and cannot be interpreted as causal. They may represent either neurodevelopmental causes underlying disease onset or susceptibility (Grotzinger et al., 2025), or secondary outcomes arising from disease progression, treatment, or functional compensation (Aguirre et al., 2025). Future prospective cohort studies tracking clinically high-risk populations (Wannan et al., 2025; Wannan et al., 2024) or longitudinal intervention studies in patients (Roell et al., 2025) may clarify whether these features constitute the cause or the effect of the disorder.

Multimodal features of structural and functional MRI of the human brain play a key role in the diagnosis of psychiatric disorders (Qureshi et al., 2017; Odusami et al., 2024). Looking forward, researchers can combine the mean amplitude features of brain regions in DMD modes with effective features of other structures or modes to achieve a more accurate diagnosis. By fusing multimodal MRI data, artificial intelligence and data-driven techniques, we have the potential to develop more accurate diagnostic tools for schizophrenia imaging assistance. Not only can these tools enhance diagnostic accuracy, but future studies can build on these findings to further explore the mechanisms by which specific brain regions play a role in schizophrenia and how they are linked to disease symptoms and progression. In addition, the abnormal signaling characteristics of these brain regions may also serve as a basis for the development of new pharmacological and non-pharmacological treatments, thus providing more precise and individualized treatment options for schizophrenia patients.

## 5 Conclusion

In this paper, our results highlight the effectiveness of DMD in extracting mean amplitudes of modes of brain regions from resting-state fMRI data as features for probing biomarkers in schizophrenia patients, and the use of LASSO regression to discover abnormal brain regions in patients, as well as the unique advantage of capturing and frequency-dependent abnormal brain region signals. As a data-driven equation-free algorithm, DMD provides an explicit representation of the time-dependent changes of the BOLD signal within a certain time window, which is well suited for extracting features and analyzing dynamic spatio-temporal patterns in fMRI data, and is a promising tool in future neuroscience research.

## Data Availability

Publicly available datasets were analyzed in this study. This data can be found at: https://fcon_1000.projects.nitrc.org/indi/retro/cobre.html.

## Footnotes

1
http://fcon_1000.projects.nitrc.org/indi/retro/cobre.html
