# Supplementary material for: Dynamic mode decomposition of resting-state fMRI revealing abnormal brain region features in schizophrenia
Source: Front Comput Neurosci. 2026 Jan 14;19:1742563. doi: 10.3389/fncom.2025.1742563 (PMC12847263; doi:10.3389/fncom.2025.1742563)
Supplement: Supplementary file 1 [file Data_Sheet_1.docx]

Supplementary Material

# Additional analyses of the results of the 300 brain regions template used in the main text


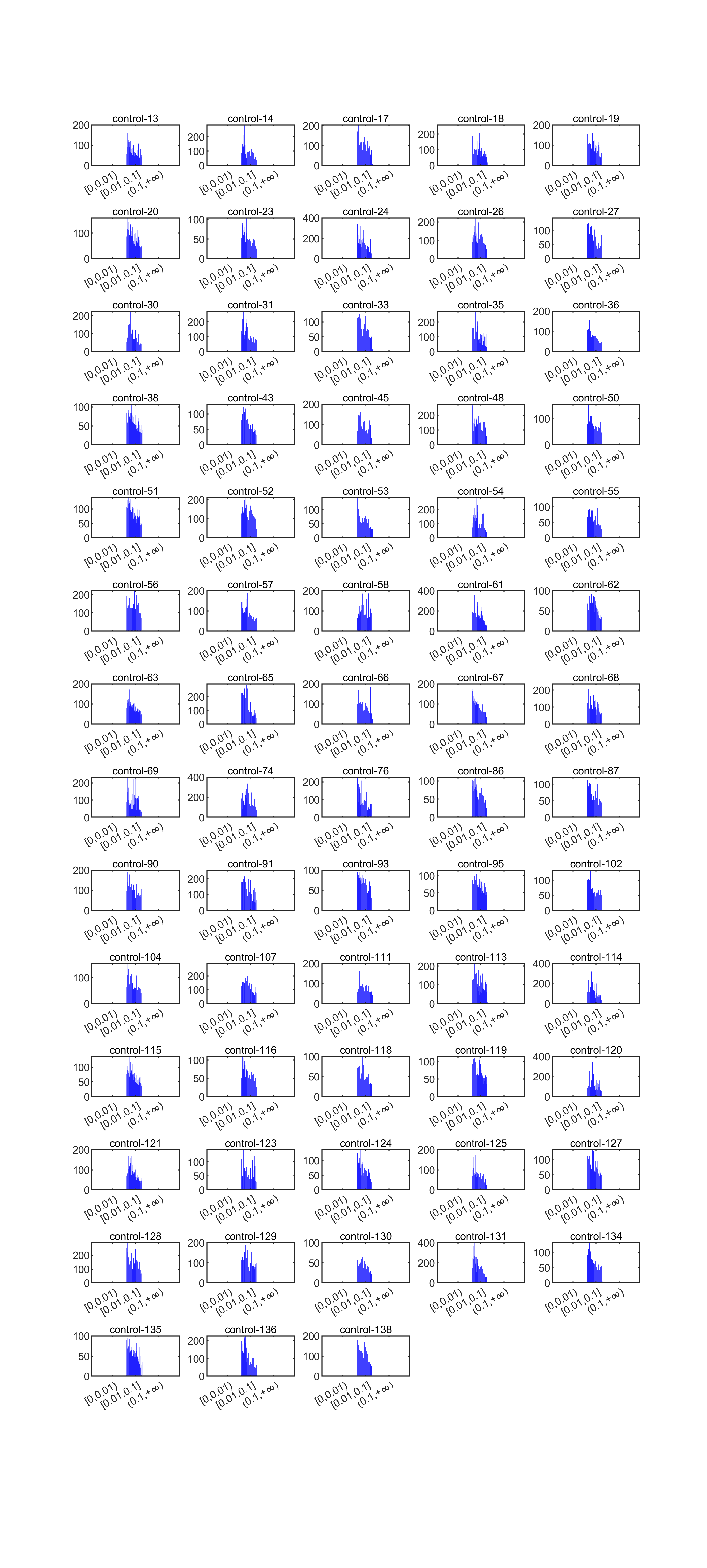


**Supplementary Figure 1.** Mode amplitude distributions in three frequency bands for 68 health subjects. The horizontal coordinates indicate the three frequency bands, $[\mathbf{0},\mathbf{0}.\mathbf{01})$, $[\mathbf{0}.\mathbf{01},\mathbf{0}.\mathbf{1}]$, and $(\mathbf{0}.\mathbf{1},+\infty)$, and the vertical coordinates indicate the magnitude of the mode amplitude.


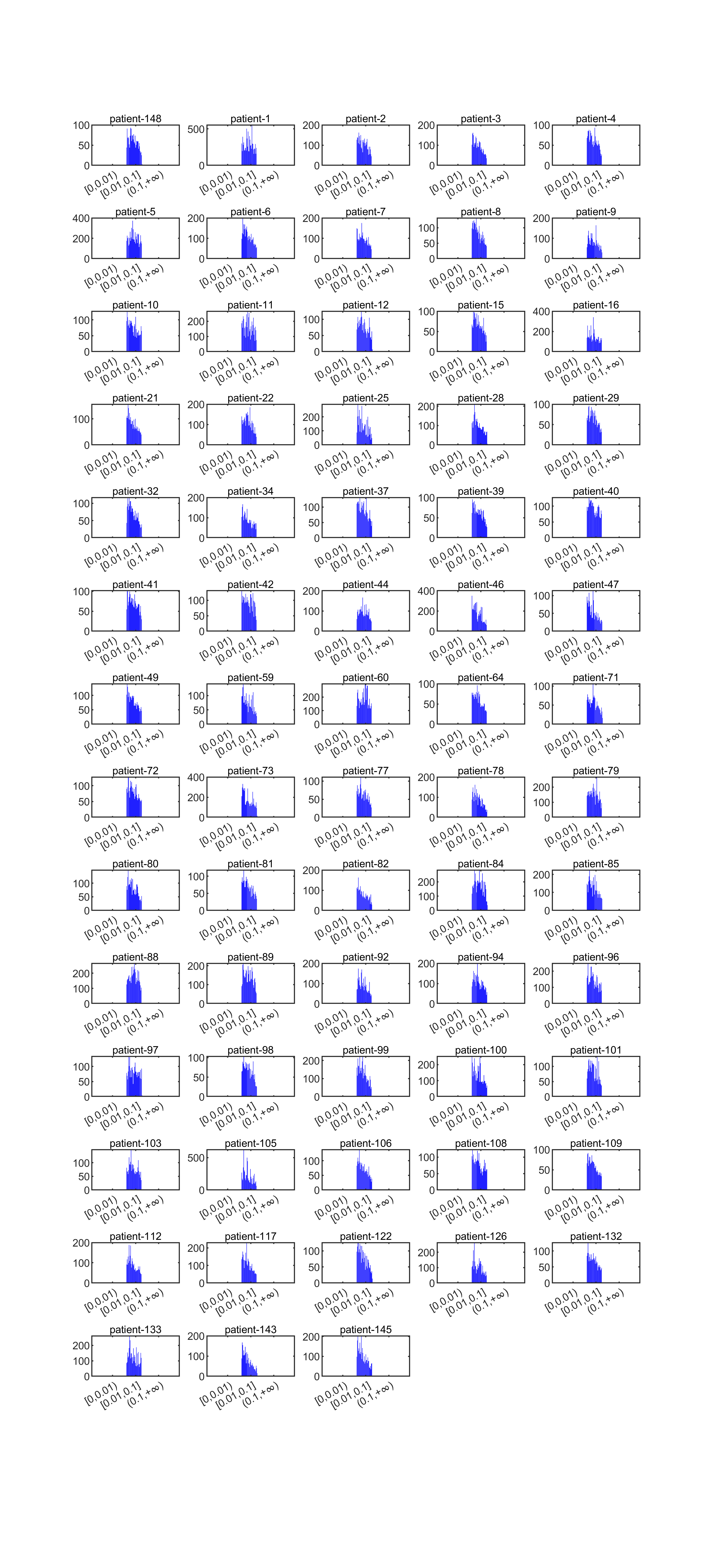


**Supplementary Figure 2.** Mode amplitude distributions in three frequency bands for 68 patients with schizophrenia. The horizontal coordinates indicate the three frequency bands, $[\mathbf{0},\mathbf{0}.\mathbf{01})$, $[\mathbf{0}.\mathbf{01},\mathbf{0}.\mathbf{1}]$, and$(\mathbf{0}.\mathbf{1},+\infty)$, and the vertical coordinates indicate the magnitude of the mode amplitude.

## Calculating Gram Matrix and snDM Features of BOLD Signals using Raw Features Extracted by DMD, PCA, ICA

In order to verify the validity of the mean amplitudes of brain regions extracted by DMD as features, we used several methods of extracting features for comparative analysis. Specifically, in addition to the original features extracted using DMD (i.e., the decomposed eigenvector matrix $\text{ϕ}$), we also decomposed the transpose of BOLD signals using PCA and ICA. In PCA, we computed the covariance matrix of the input data and performed eigenvalue decomposition of the covariance matrix to select a certain number of eigenvectors corresponding to the largest eigenvalues as principal component matrices; in ICA, we whitened the input data and searched for the directions which make the signal non-Gaussian to be maximized by an iterative optimization algorithm, using these directions as the independent component matrices. The principal component matrix $\text{F}_{\text{1}}$ and independent component matrix $\text{F}_{\text{2}}$ obtained after decomposition were considered as the original features extracted by PCA and ICA, respectively, and the corresponding Gram matrices were calculated based on $\text{ϕ, }\text{F}_{\text{1}}\text{, }\text{F}_{\text{2}}$, which represents the similarity of original features between subjects and can also be used as an input to SVM.

Eq. (1) calculates the Gram matrix between subjects *k* and *l* using $\text{ϕ}$ as an example. The number of features extracted by the three different methods of DMD, PCA, and ICA were set as follows: DMD - each subject retains the number of all modes obtained by decomposition within 0.01-0.1 Hz; PCA - each subject retains the minimum number of features when the cumulative variance reaches 100%; ICA - each subject retains the maximum number of independent components that can be obtained by decomposition. This setting can preserve as much information about the original data as possible and also reduce data redundancy. For the computed Gram matrix $\text{G}$ of dimension *N×N* (N=136), we divide all the elements of the matrix by the average of all the elements as the extracted Gram matrix features $\overline{\text{G}}$ (defined in (2)).

In addition, we investigated the validity of the spatial node DMD features (snDM), which can capture cortical activity similar to high gamma power features. Multiplying the $\text{ϕ}$ obtained by DMD with its conjugate transpose $\text{ϕ}^{\text{*}}$ yields a symmetric matrix $\text{ϕ}\text{ϕ}^{\text{*}}$ with a dimension of *m×m* (m=300) and we expanded the diagonal elements of $\text{ϕ}\text{ϕ}^{\text{*}}$ into a vector of length *m=300* (number of brain regions), which was used as the snDM features for each subject.

$\text{G}_{\text{kl}}\text{=}\sum_{\text{i=1}}^{\text{M}} {\sum_{\text{j=1}}^{\text{M}} \text{|}\text{φ}_{\text{ki}}^{\text{*}}\text{φ}_{\text{lj}}\text{|}}^{\text{2}}\text{, }\text{k,l}\text{=1…N}$ (1)

$\text{G=}{\text{(}\text{G}_{\text{kl}}\text{)}}_{\text{N×N}}\text{, }\overline{\text{G}}\text{=}\frac{\text{G}}{\frac{\text{1}}{\text{N×N}}\sum_{\text{i=1}}^{\text{N}} \sum_{\text{j=1}}^{\text{N}} \text{G}_{\text{ij}}}$ (2)

The results (Supplementary Figure 3) showed that the classification performance of DMD-LASSO did not differ significantly from that of DMD-Gram, PCA-Gram, or snDM-LASSO, whereas all four methods (DMD-LASSO, DMD-Gram, PCA-Gram, and snDM-LASSO) significantly outperformed ICA-Gram.


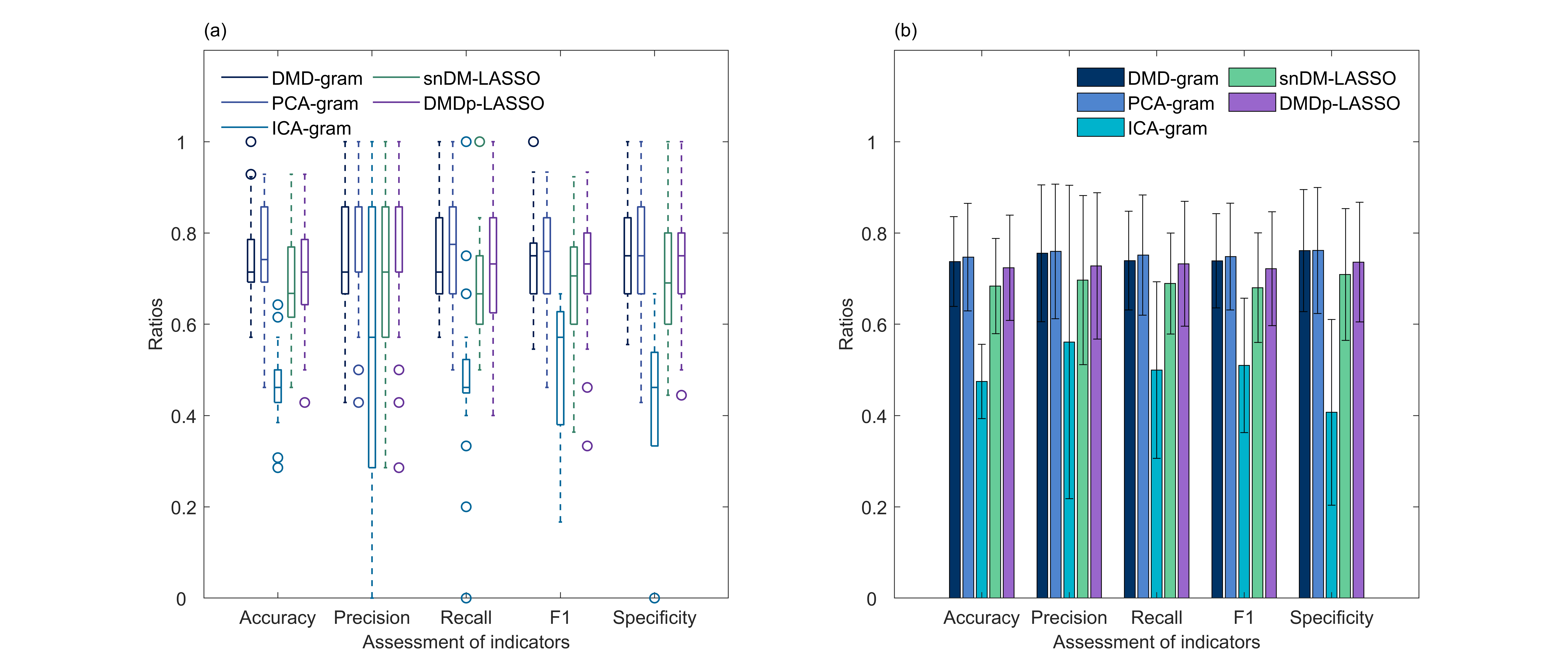


**Supplementary Figure 3.** The classification performance of DMD-LASSO did not differ significantly from that of DMD-Gram, PCA-Gram, or snDM-LASSO. (a) Box plots and (b) mean bar plots of linear SVM classification indicators across the five methods.

**Supplementary Table 1.** Permutation Test P-values for the Linear SVM Classification Indicators for DMD-gram, PCA-gram, ICA-gram, snDM-LASSO, and DMDp-LASSO

| Objects of the permutation test  (p_value) | Accuracy | Precision | Recall | F1 | Specificity |
| --- | --- | --- | --- | --- | --- |
| DMDp-LASSO vs DMD-gram | 6.02E-01 | 4.70E-01 | 8.21E-01 | 5.74E-01 | 4.45E-01 |
| DMDp-LASSO vs PCA-gram | 4.63E-01 | 4.15E-01 | 5.89E-01 | 3.99E-01 | 4.70E-01 |
| DMDp-LASSO vs ICA-gram | 9.99E-04*** | 1.60E-02* | 9.99E-04*** | 9.99E-04*** | 9.99E-04*** |
| DMDp-LASSO vs snDM-LASSO | 1.62E-01 | 4.82E-01 | 1.87E-01 | 1.91E-01 | 4.49E-01 |
| DMD-gram vs PCA-gram | 7.28E-01 | 9.23E-01 | 7.00E-01 | 7.51E-01 | 9.93E-01 |
| DMD-gram vs ICA-gram | 9.99E-04*** | 1.10E-02* | 9.99E-04*** | 9.99E-04*** | 9.99E-04*** |
| DMD-gram vs snDM-LASSO | 4.20E-02* | 1.87E-01 | 8.49E-02 | 4.20E-02* | 1.66E-01 |
| PCA-gram vs ICA-gram | 9.99E-04*** | 9.99E-03** | 9.99E-04*** | 9.99E-04*** | 9.99E-04*** |
| PCA-gram vs snDM-LASSO | 3.90E-02* | 1.49E-01 | 6.89E-02 | 2.90E-02* | 1.43E-01 |
| ICA-gram vs snDM-LASSO | 9.99E-04*** | 5.39E-02 | 9.99E-04*** | 9.99E-04*** | 9.99E-04*** |

Note: * indicates p-value < 0.05; ** indicates p-value < 0.01; *** indicates p-value < 0.001.


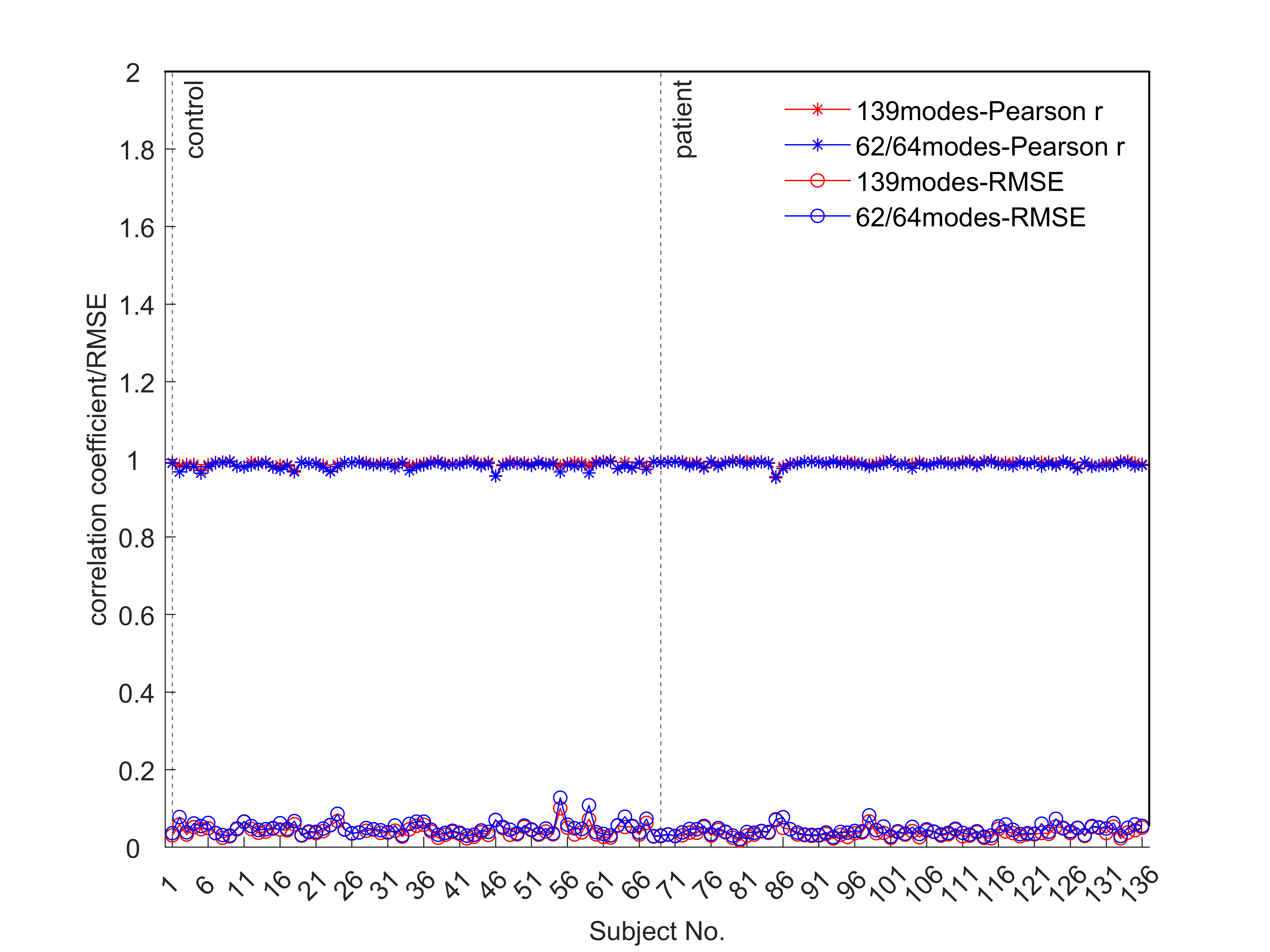


**Supplementary Figure 4.** Correlations and RMSEs between phiC and FC calculated using 139 modes and using 62/64 modes. In the figure, the horizontal coordinates represent the numbering of the subjects, with 1-68 numbered as healthy subjects and 69-136 numbered as schizophrenics.

# Replication and validation of DMD analysis using a 100 brain regions template

To test the reproducibility of the results, we used a template of 100 brain regions from ‘Schaefer2018_100Parcels_7Networks_order_FSLMNI152_2mm’ to define the regions of interest and obtained consistent results.

The DMD algorithm was originally used for research in the field of fluid dynamics in physics, where the input data matrix is usually in the form of a thin height, i.e. the number of rows of the data matrix is much greater than the number of columns. The size of the time series matrix of the BOLD signal obtained using the 100 brain region template is $100\times140$ (where 100 is the number of brain regions and 140 is the number of time points), which after transposition is the data matrix input to the DMD, and the number of columns of the data matrix is more than the number of rows, and this feature will limit the maximum number of modes and singular values of the dynamic mode decomposition, and the dynamics of the original matrix can't be fully captured. Therefore, for the BOLD signal time series obtained from the 100 brain regions template, we used the data matrix augmentation method to implement the DMD algorithm.

The data augmentation method assumes that the original data matrix is $X_{n\times m}$, and the number of measurements is increased to $h*n$ by appending a matrix of measurements with $h$ time offsets to the measurements at the sampling points. That is, the original data matrix $X$ is reconstructed as $X_{aug}$:

$$X=\left[\begin{matrix} | & | & & | \\ x_{1} & x_{2} & ... & x_{m} \\ | & | & & | \end{matrix}\right]\Rightarrow X_{aug}=\left[\begin{matrix} | & | & & | \\ x_{1} & x_{2} & ... & x_{m-h\text{+1}} \\ | & | & & | \\ | & | & & | \\ x_{2} & x_{3} & ... & x_{m-h+2} \\ | & | & & | \\ & & \vdots& \\ | & | & & | \\ x_{h} & x_{h+1} & & x_{m} \\ | & | & & | \end{matrix}\right]$$

When the DMD algorithm is applied to the new enhanced data matrix, the resulting $\phi$ also becomes a matrix of the size of $(h*n)\times m$. However, since the new enhanced matrix is a stack of $h$ time offsets of the same original data, the computed $\phi$ is also a repeated stack of $h$ data matrices, and it is sufficient to extract the first layer of the $\phi$ for subsequent investigation and analysis. Furthermore, it was found that the value of the stacking depth parameter $h$ is generally chosen to be the smallest integer such that $h*n>2*m$. The size of the data matrix corresponding to the 100 brain regions templates is n=100, m=140, so the stacking depth parameter chosen is h=3. This means that we append the time offsets of 3 sampling points to the matrix of measurements, thus increasing the number of measurements (i.e., the length of the matrix) to 300, and the resulting eigenvector matrix is decomposed by taking only the first layer (the first 100 rows) for subsequent analysis.


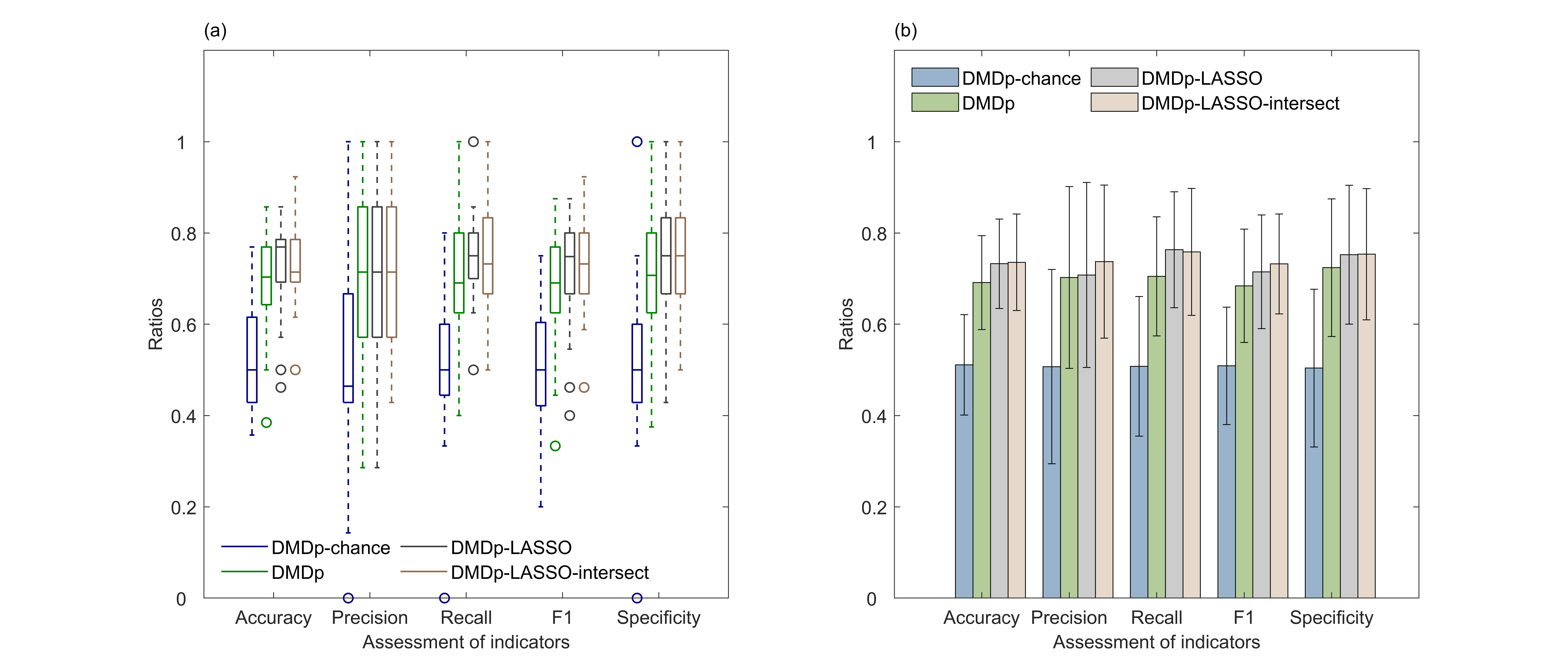


**Supplementary Figure 5.** Using DMDp, DMDp-LASSO, DMDp-LASSO-intersect as classification features were significantly better than DMDp-chance. (a) Box plots and (b) mean bar plots of linear SVM classification indicators for the four compared cases.

**Supplementary Table 2.** Permutation Test P-values for the Linear SVM Classification Indicators for DMDp-chance, DMDp, DMDp-LASSO and DMDp-LASSO-intersect

| Objects of the permutation test  (p_value) | Accuracy | Precision | Recall | F1 | Specificity |
| --- | --- | --- | --- | --- | --- |
| DMDp vs DMDp-chance | 9.90E-03** | 9.90E-03** | 9.90E-03** | 9.90E-03** | 9.90E-03** |
| DMDp vs DMDp-LASSO | 1.29E-01 | 8.71E-01 | 1.09E-01 | 3.96E-01 | 4.85E-01 |
| DMDp vs DMDp-LASSO-intersect | 1.78E-01 | 5.05E-01 | 1.09E-01 | 1.39E-01 | 3.56E-01 |
| DMDp-LASSO vs DMDp-chance | 9.90E-03** | 9.90E-03** | 9.90E-03** | 9.90E-03** | 9.90E-03** |
| DMDp-LASSO vs DMDp-LASSO-intersect | 9.01E-01 | 5.84E-01 | 8.91E-01 | 6.04E-01 | 9.80E-01 |
| DMDp-LASSO-intersect vs DMDp-chance | 9.90E-03** | 9.90E-03** | 9.90E-03** | 9.90E-03** | 9.90E-03** |

Note: * indicates p-value < 0.05; ** indicates p-value < 0.01; *** indicates p-value < 0.001.


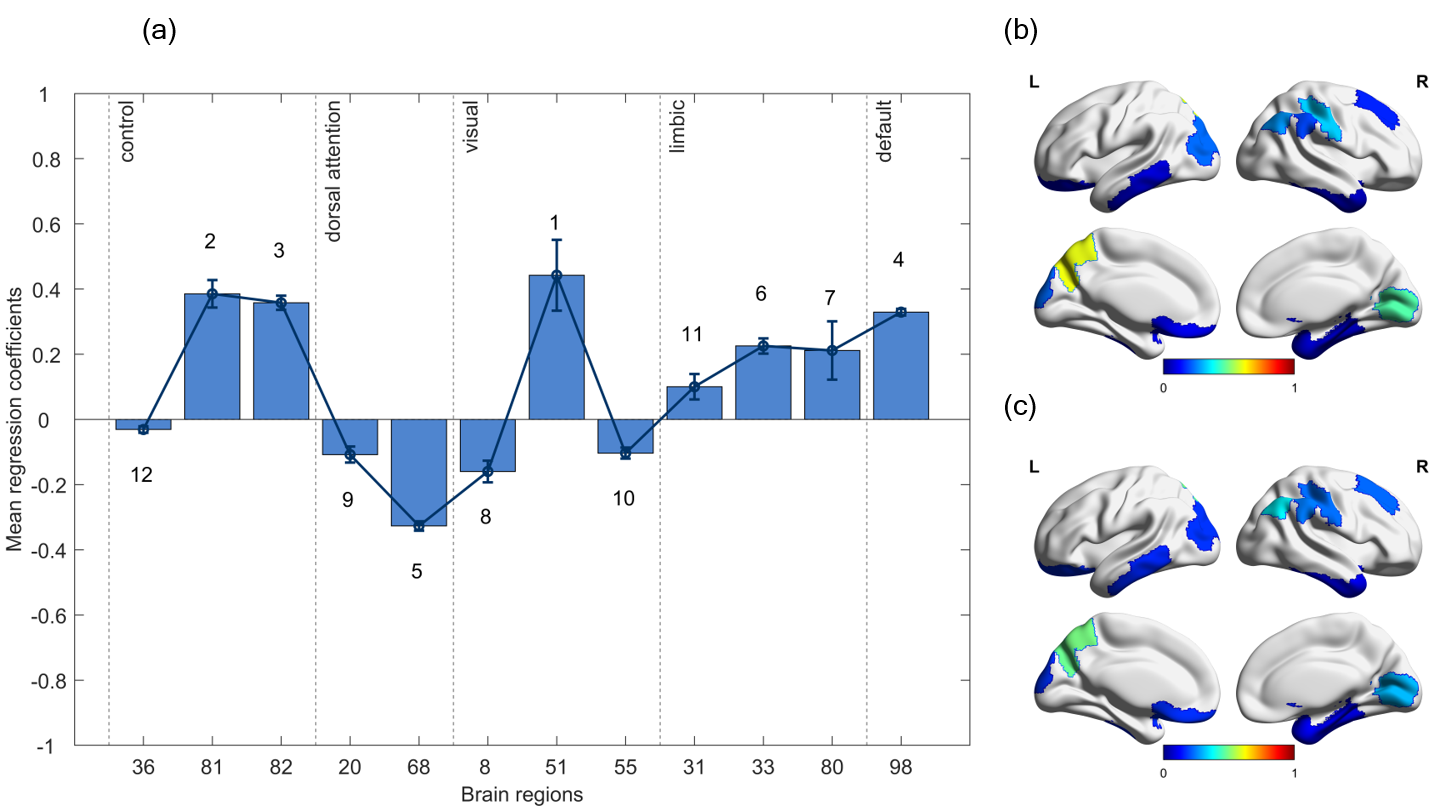


**Supplementary Figure 6.** Brain regions in several functional networks had the same direction of deviations of abnormality. (a) Mean regression coefficients for abnormal brain region features in the full frequency band. The associated number ranks each feature's importance for classification (1 = most influential), based on $\text{|}\overline{\text{β}_{\text{i}}}\text{|}$ (mean absolute regression coefficient). (b-c) Visualization of the mean amplitudes for the 12 abnormal brain regions in (b) the healthy group and (c) schizophrenia patients.


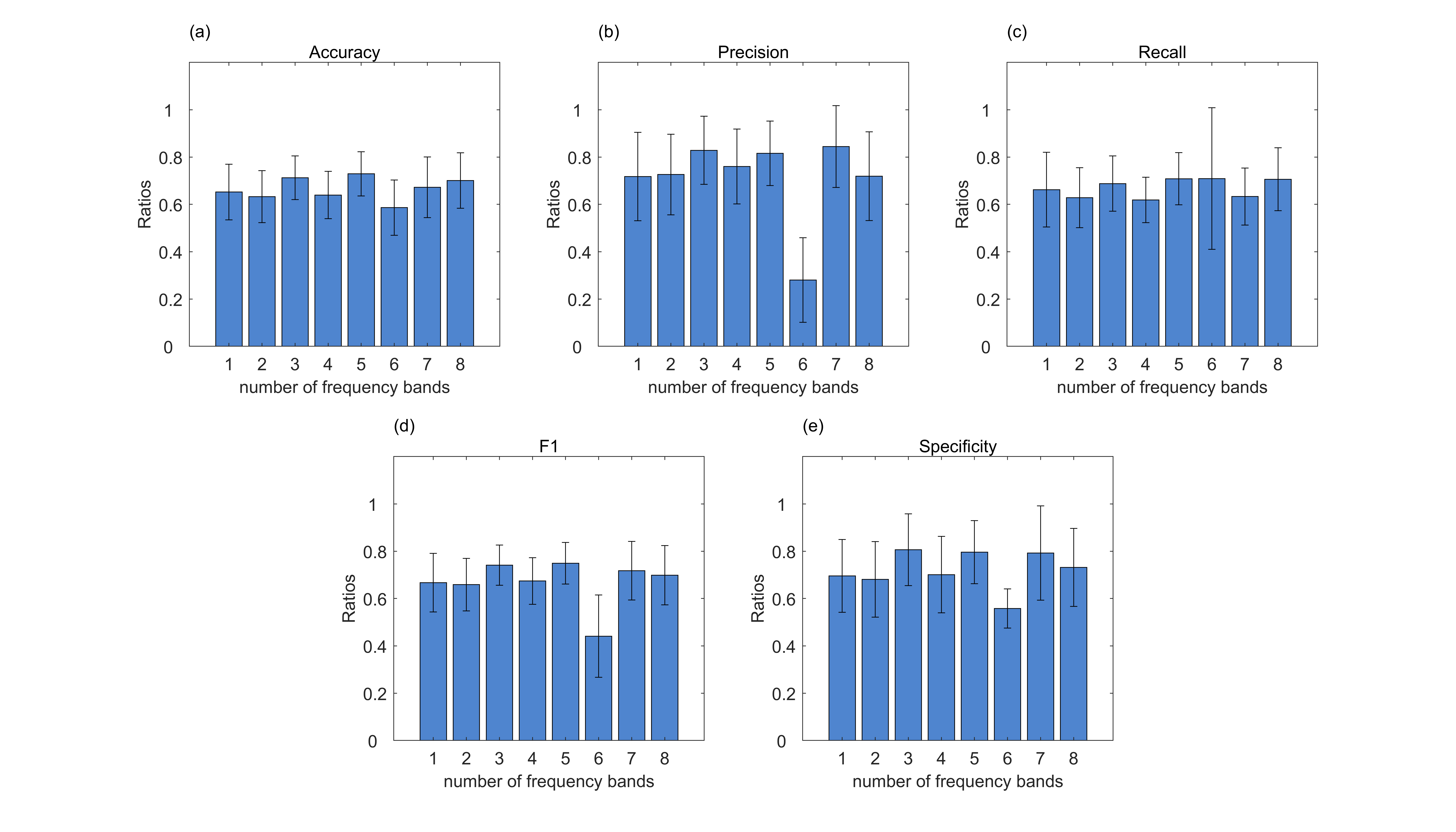


**Supplementary Figure 7.** Classification effects can be significantly improved by using mean amplitude features of brain region modes averaged over three frequency bands. (a)-(e) shows the bar graphs of frequency band comparison for the five indicators. The horizontal coordinates indicate the number of frequency bands, in particular, 1 indicates that there is only one band, i.e., no sub-banding.


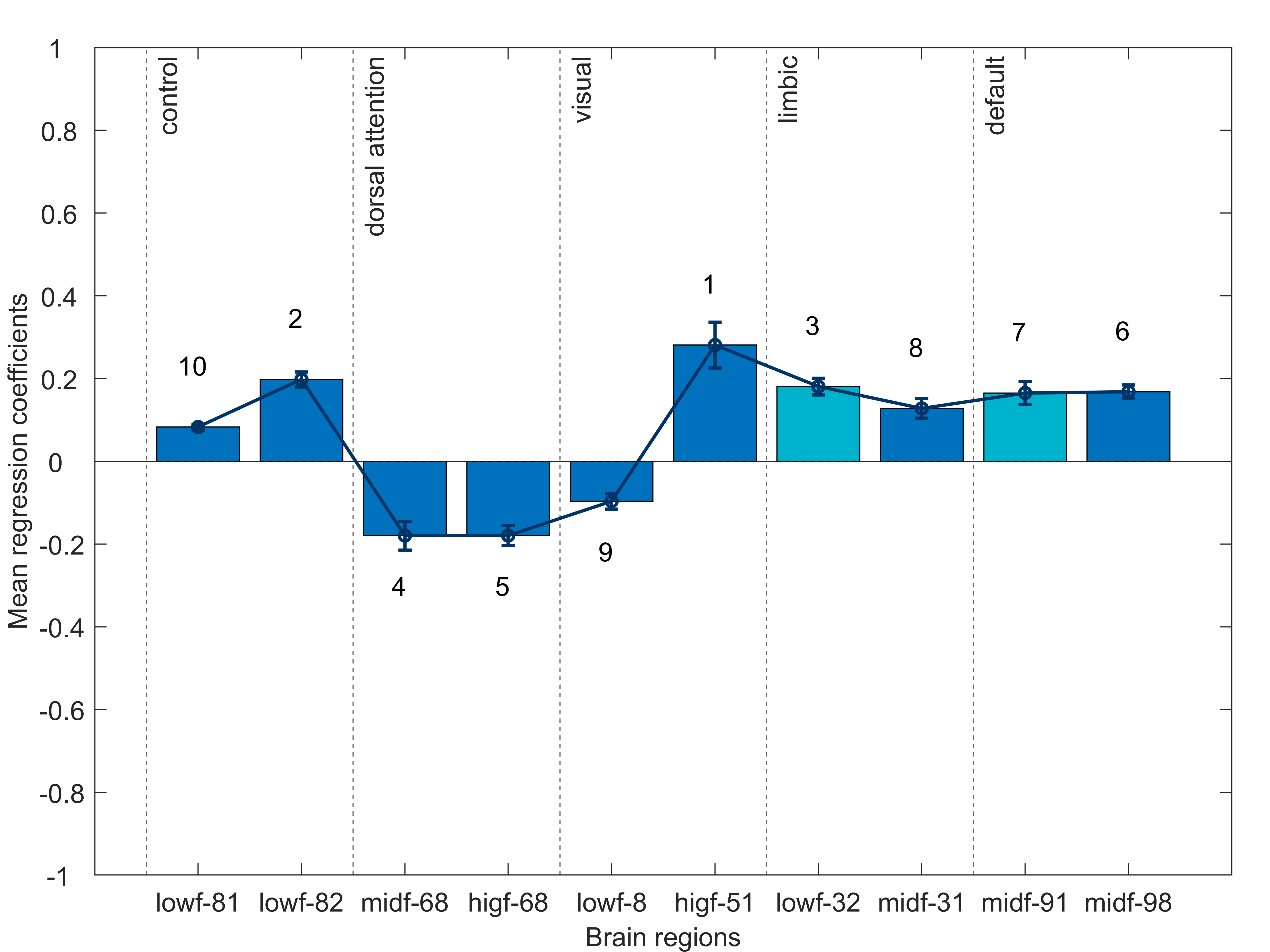


**Supplementary Figure 8.** After subdividing the frequency bands, several newly identified abnormal brain region features were detected in the limbic network and default network. Bar graph of the mean regression coefficients for features identified within three sub-bands: low- (lowf, [0.01,0.04) Hz), mid- (midf, [0.04,0.07) Hz), and high-frequency (higf, [0.07,0.1] Hz). Features highlighted in cyan were newly detected and not found in the full-band analysis.


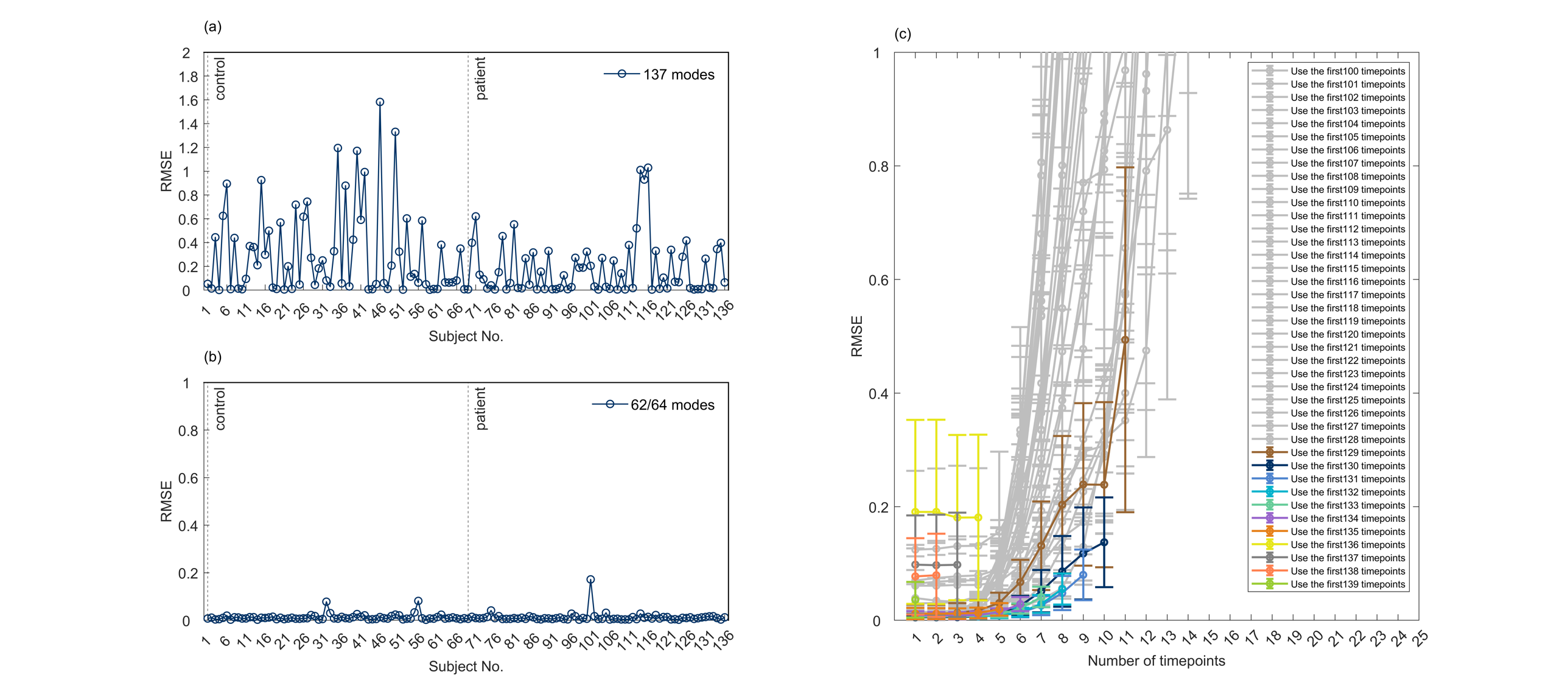


**Supplementary Figure 9.** DMD enables effective BOLD signal reconstruction and prediction. (a–b) Reconstruction performance using (a) all decomposed modes versus (b) noise-filtered modes per subject. (c) Mean prediction error (RMSE) versus the number of future time points forecasted, based on decompositions starting from varying initial segments of the time series.


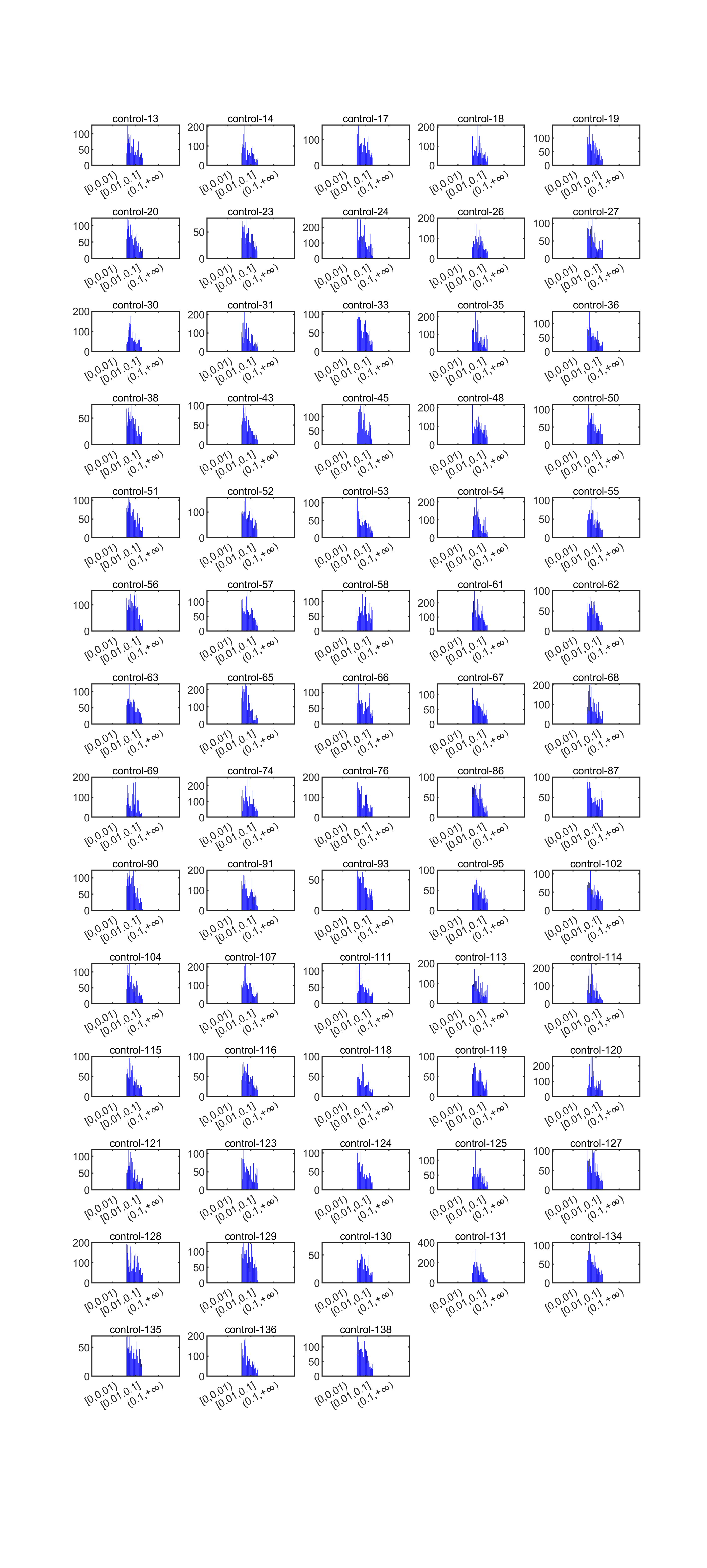


**Supplementary Figure 10.** Mode amplitude distributions in three frequency bands for 68 health subjects. The horizontal coordinates indicate the three frequency bands, $[\mathbf{0},\mathbf{0}.\mathbf{01})$, $[\mathbf{0}.\mathbf{01},\mathbf{0}.\mathbf{1}]$, and $(\mathbf{0}.\mathbf{1},+\infty)$, and the vertical coordinates indicate the magnitude of the mode amplitude.


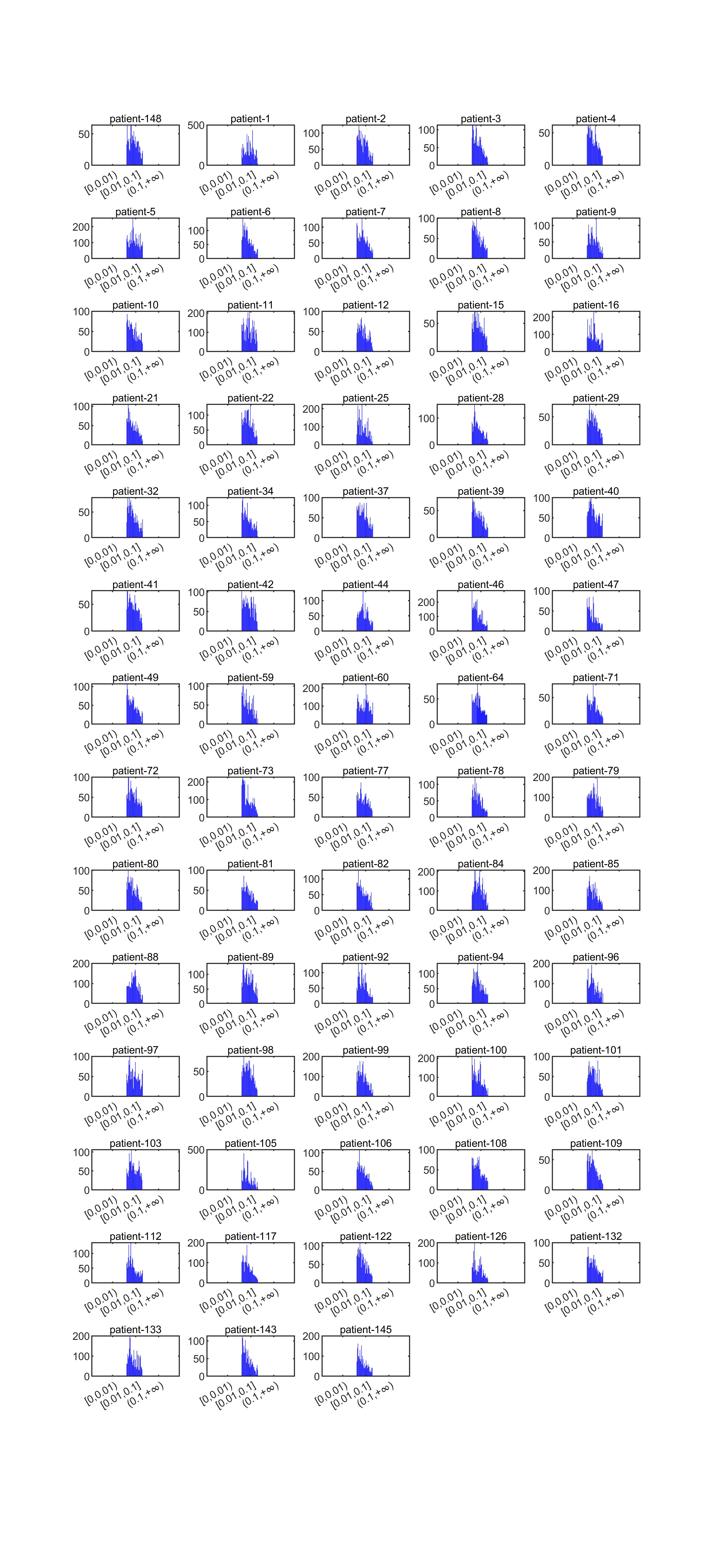


**Supplementary Figure 11.** Mode amplitude distributions in three frequency bands for 68 patients with schizophrenia. The horizontal coordinates indicate the three frequency bands, $[\mathbf{0},\mathbf{0}.\mathbf{01})$, $[\mathbf{0}.\mathbf{01},\mathbf{0}.\mathbf{1}]$, and$(\mathbf{0}.\mathbf{1},+\infty)$, and the vertical coordinates indicate the magnitude of the mode amplitude.


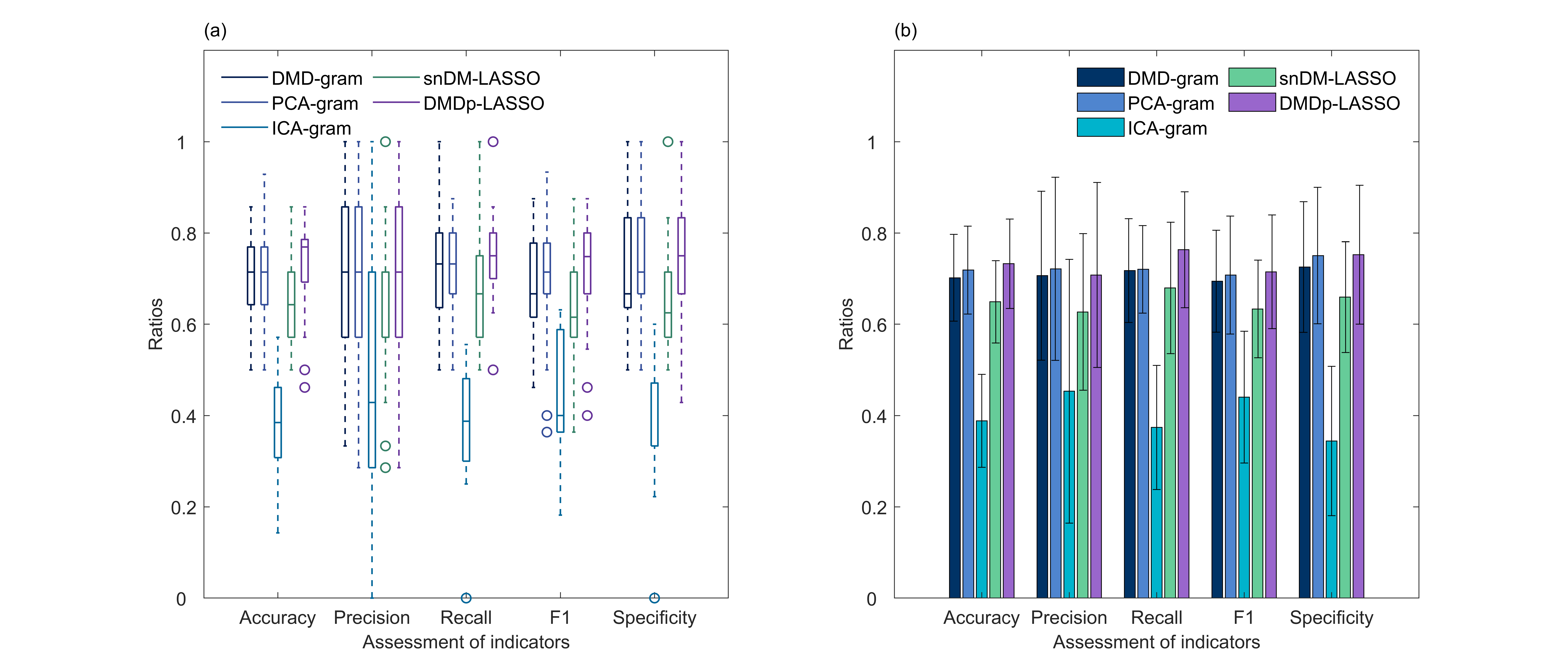


**Supplementary Figure 12.** The classification performance of DMD-LASSO did not differ significantly from that of DMD-Gram and PCA-Gram, but was significantly better than ICA-Gram and snDM-LASSO. (a) Box plots and (b) mean bar plots of linear SVM classification indicators across the five methods.

**Supplementary Table 3.** Permutation Test P-values for the Linear SVM Classification Indicators for DMD-gram, PCA-gram, ICA-gram, snDM-LASSO, and DMDp-LASSO

| Objects of the permutation test  (p_value) | Accuracy | Precision | Recall | F1 | Specificity |
| --- | --- | --- | --- | --- | --- |
| DMDp-LASSO vs DMD-gram | 2.58E-01 | 9.81E-01 | 1.41E-01 | 5.08E-01 | 4.85E-01 |
| DMDp-LASSO vs PCA-gram | 5.59E-01 | 7.87E-01 | 1.44E-01 | 8.35E-01 | 9.70E-01 |
| DMDp-LASSO vs ICA-gram | 9.99E-04*** | 9.99E-04*** | 9.99E-04*** | 9.99E-04*** | 9.99E-04*** |
| DMDp-LASSO vs snDM-LASSO | 2.00E-03** | 1.10E-01 | 2.00E-02* | 9.99E-03** | 1.40E-02* |
| DMD-gram vs PCA-gram | 5.10E-01 | 7.73E-01 | 9.21E-01 | 6.84E-01 | 4.81E-01 |
| DMD-gram vs ICA-gram | 9.99E-04*** | 9.99E-04*** | 9.99E-04*** | 9.99E-04*** | 9.99E-04*** |
| DMD-gram vs snDM-LASSO | 3.10E-02* | 9.29E-02 | 2.57E-01 | 3.50E-02* | 5.00E-02* |
| PCA-gram vs ICA-gram | 9.99E-04*** | 2.00E-03** | 9.99E-04*** | 9.99E-04*** | 9.99E-04*** |
| PCA-gram vs snDM-LASSO | 6.99E-03** | 6.19E-02 | 2.05E-01 | 1.50E-02* | 5.00E-03** |
| ICA-gram vs snDM-LASSO | 9.99E-04*** | 5.99E-03** | 9.99E-04*** | 9.99E-04*** | 9.99E-04*** |

Note: * indicates p-value < 0.05; ** indicates p-value < 0.01; *** indicates p-value < 0.001.


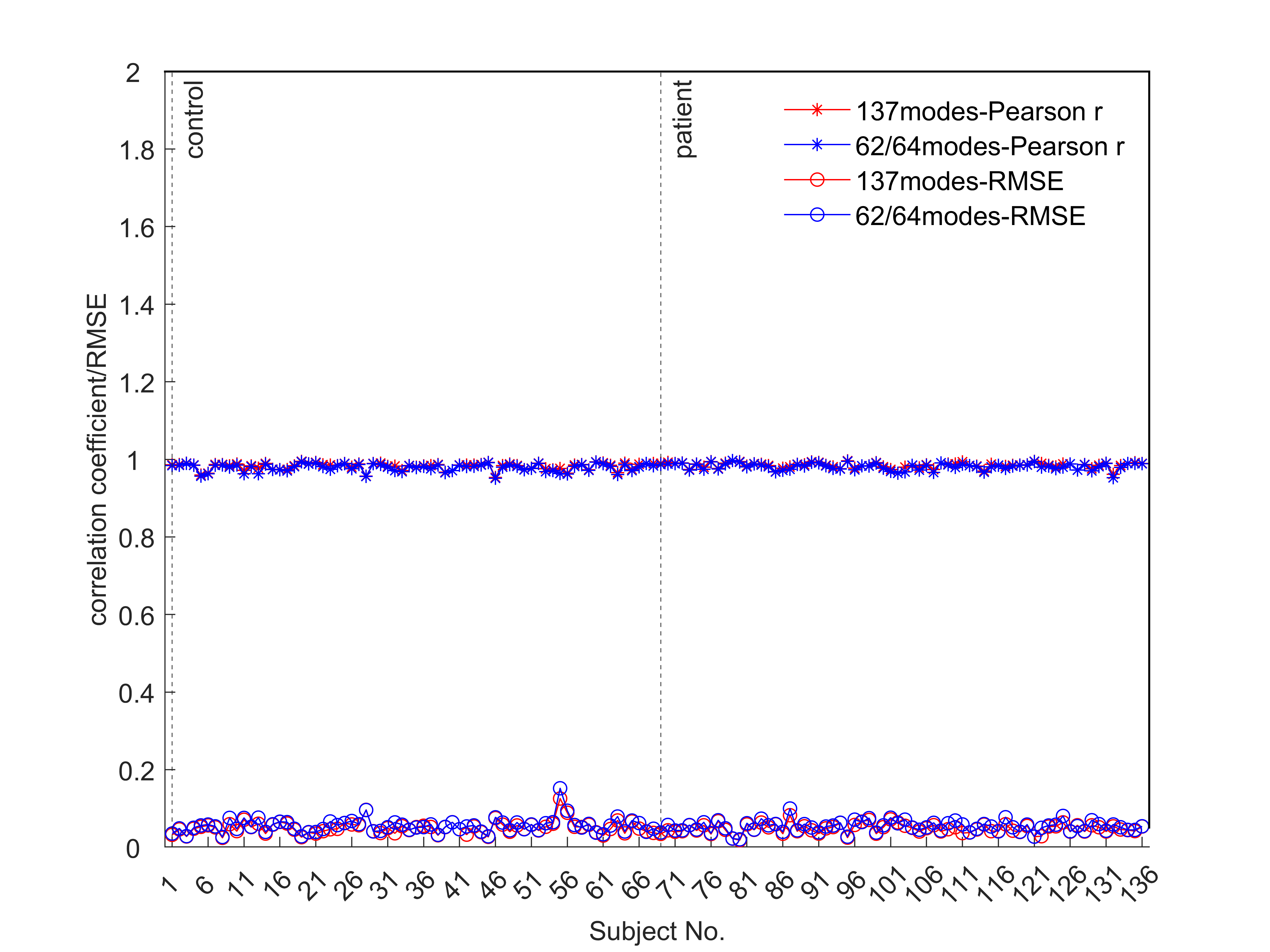


**Supplementary Figure 13.** Correlations and RMSEs between phiC and FC calculated using 137 modes and using 62/64 modes. In the figure, the horizontal coordinates represent the numbering of the subjects, with 1-68 numbered as healthy subjects and 69-136 numbered as schizophrenics.
